# Near infrared photoimmunotherapy of cancer; possible clinical applications

**DOI:** 10.1515/nanoph-2021-0119

**Published:** 2021-05-07

**Authors:** Hiroaki Wakiyama, Takuya Kato, Aki Furusawa, Peter L. Choyke, Hisataka Kobayashi

**Affiliations:** Molecular Imaging Branch, Center for Cancer Research, National Cancer Institute, NIH, Bethesda, MD, 20892, USA

**Keywords:** anti-cancer host immunity, cancer, immunogenic cell death, near-infrared photoimmunotherapy (NIR-PIT), super-enhanced permeability and retention (SUPR) effects

## Abstract

Near-infrared photoimmunotherapy (NIR-PIT) is a new cancer treatment that uses an antibody-photo-absorber conjugate (APC) composed of a targeting monoclonal antibody conjugated with a photoactivatable phthalocyanine-derivative dye, IRDye700DX (IR700). APCs injected into the body can bind to cancer cells where they are activated by local exposure to NIR light typically delivered by a NIR laser. NIR light alters the APC chemical conformation inducing damage to cancer cell membranes, resulting in necrotic cell death within minutes of light exposure. NIR-PIT selectivity kills cancer cells by immunogenic cell death (ICD) with minimal damage to adjacent normal cells thus, leading to rapid recovery by the patient. Moreover, since NIR-PIT induces ICD only on cancer cells, NIR-PIT initiates and activates antitumor host immunity that could be further enhanced when combined with immune checkpoint inhibition. NIR-PIT induces dramatic changes in the tumor vascularity causing the super-enhanced permeability and retention (SUPR) effect that dramatically enhances nanodrug delivery to the tumor bed. Currently, a worldwide Phase 3 study of NIR-PIT for recurrent or inoperable head and neck cancer patients is underway. In September 2020, the first APC and accompanying laser system were conditionally approved for clinical use in Japan. In this review, we introduce NIR-PIT and the SUPR effect and summarize possible applications of NIR-PIT in a variety of cancers.

## 1 Introduction

Cancer is the second leading cause of death globally [1]. It is estimated that 19.3 million new cancer cases and almost 10.0 million cancer deaths occurred in 2020. Moreover, cancer incidence and mortality is rapidly rising worldwide, reflecting aging and environmental exposures [2]. Three major cancer therapies; surgery, radiation therapy, and chemotherapy have been the mainstay of cancer treatment for many decades. Each method can reduce cancer burden, however, each treatment also causes severe collateral damage to normal cells including immune cells and stem cells contributing to disease recurrence and delayed healing, and resulting in significant consequences for quality of life. In the last decade, improved cancer immunotherapies have dramatically altered the therapeutic landscape [3]. However, the effectiveness of immunotherapy depends on altering the careful balance of effector T cells and immune suppressor cells [4]. Although it can produce spectacular results, the overall response rate of immunotherapy remains relatively low, mostly because of the absence of T cell infiltration in tumors [5]. Meanwhile, immunotherapy-related side effects, termed immune-related adverse events (irAEs), have been widely reported and often mimic autoimmune disease. It has been reported that irAEs were observed in up to 90% of patients treated with an anti-cytotoxic T-lymphocyte antigen 4 (CTLA-4) drug and 70% of those treated with a programmed death-1 (PD-1)/PD-ligand 1 (PD-L1) inhibitor, two common checkpoint inhibitors [6]. Thus, despite advances, no cancer treatment is capable of selectively killing cancer cells while activating the local host immune response. Near-infrared photoimmunotherapy (NIR-PIT) is proposed as a method to overcome these challenges.

In this review, we first provide an overview of NIR-PIT. Then, we describe how NIR-PIT can enhance nanodrug delivery based on the super-enhanced permeability and retention (SUPR) effect. Finally, we discuss possible clinical applications of NIR-PIT and SUPR to cancers arising in various organs.

## 2 NIR-PIT

**Figure 1:**
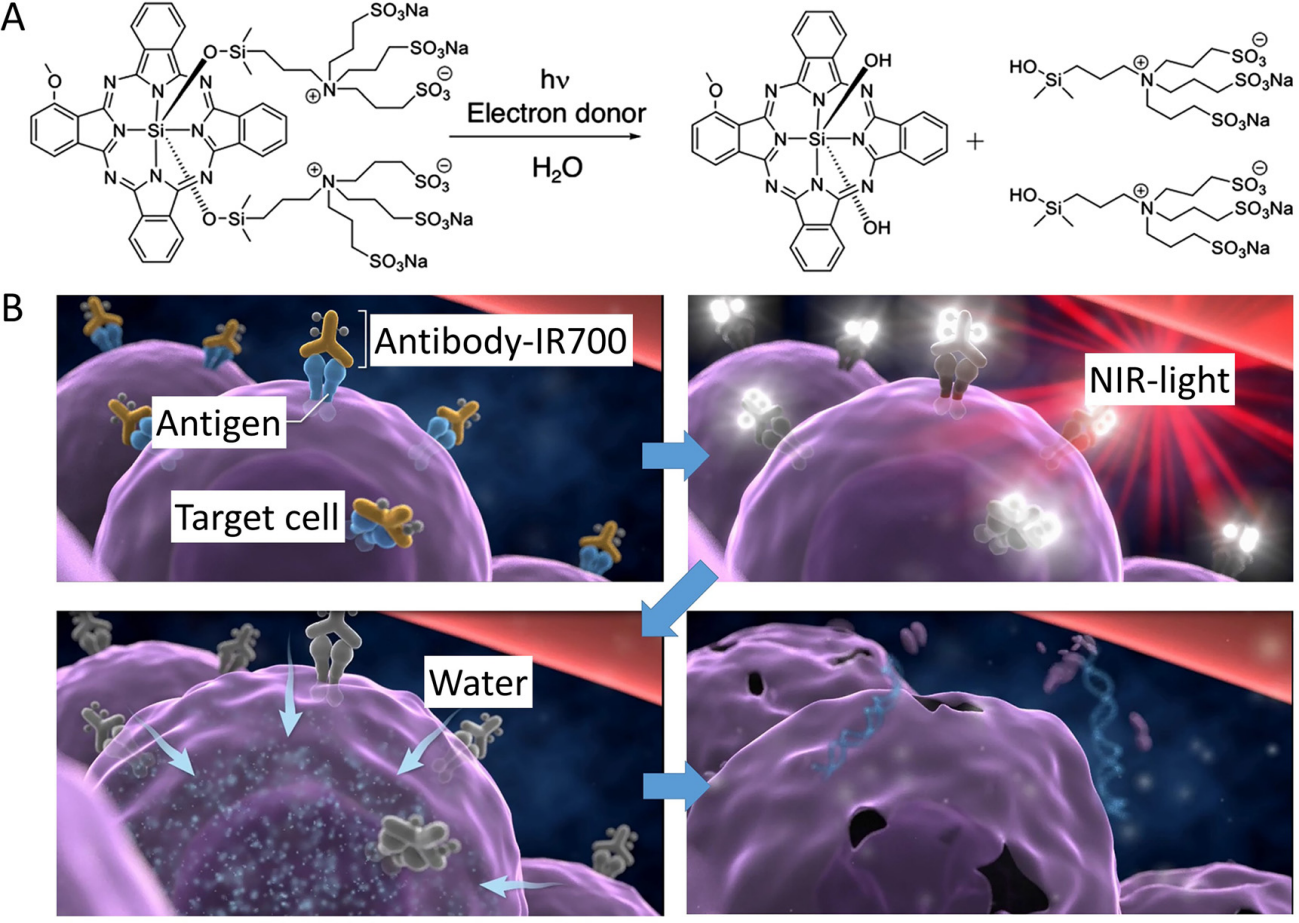
The mechanisms of cell death caused by NIR-PIT. (A) Structural change of IR700. Upon NIR light exposure, axial ligands are released from the IR700 molecule. Adapted from Ref. [8]. (B) Scheme of the cell killing mechanism induced by NIR-PIT. An antibody–IR700–antigen complex is formed on the antigen on the cell membrane. The conformational change of conjugate produces physical stress in the cell membrane, resulting in the weakening and rupture of the cell membrane. The water outside of the cell is flown into the cell, leading cell death. Adapted from Ref. [8].

NIR-PIT is a newly developed cancer treatment that employs an antibody conjugated with the NIR light-absorbing silicon phthalocyanine dye, IRDye700DX (IR700) [7], [8]. This antibody-photo-absorber conjugate (APC) is injected intravenously where it binds to specific cancer cells expressing the appropriate antigen on the cell membrane. NIR light (∼690 nm) is then directed to the tumor site activating the APC to induce cell killing [7, 9–11]. Recently the mechanism of cytotoxicity of NIR-PIT has been explained [12]. Immediately after NIR light exposure, axial ligands of the IR700 molecule, which are responsible for its hydrophilicity, are dissociated from the main molecule causing the APC to change from a highly hydrophilic to a highly hydrophobic compound (Figure 1A). This change in chemical properties of the APC promotes aggregation leading to damage and rupture of the cellular membrane. The cell membrane is progressively weakened, microperforations form and ultimately blebbing and bursting occurs, resulting in necrotic cell death (Figure 1B). Damage to the cellular membrane during NIR-PIT can be observed with such techniques as three-dimensional low-coherent quantitative phase microscopy or dual-view inverted selective plane illumination microscopy [13]. Movies of cells undergoing cell death during NIR-PIT reveal a rapid swelling of the cell, blebbing, and rupture with release of the intracellular contents into the extracellular space. This mechanism of cell death clearly distinguishes NIR-PIT from conventional photodynamic therapy (PDT), which relies on the production of reactive oxygen species to cause non selective damage to adjacent normal tissue.

In theory, NIR-PIT is most suited to treating superficial tumors because NIR light can penetrate only approximately 2 cm from the tissue surface [14]. In special circumstances, such as treating tumors in the lung and pleural cavity, NIR light can be transmitted much further through the air in the lungs [15], [16], [17], [18]. However, in more solid tissues NIR light is rapidly attenuated and thus, the light source must be placed into or nearby tumors [19]. This can be accomplished by using flexible, cylindrical, fiber optic, interstitial light diffusers that are inserted into the treatment site. Using interstitial light diffusers practically any tumor site is amenable to NIR-PIT whether inserted via needle, catheter or endoscope [20], [21]. Furthermore, implanted wireless NIR light emitting diode (LED) sources can be used to generate light repeatedly in a remote tumor site [22]. Since the IR700 is both a therapeutic and a diagnostic, fluorescence imaging can be used to detect sites of tumor to which the APC is bound and direct therapeutic doses of light to those fluorescing regions. As the light photobleaches the IR700, there is a decrease in fluorescence reaching a minimum plateau after the dye is completely photobleached. It is reported that the therapeutic effect correlates with the photo bleaching extent [12]. A commercially available camera, originally designed to image indocyanine green, which typically operates at wavelengths of 830 nm, can be repurposed to detect the low level fluorescence arising from IR700 during NIR-PIT because of the high intensity of the excitation light and the emission spectrum of IR700 which extends beyond 830 nm. This enables NIR-PIT to be monitored in real time at wavelengths far from the intense laser excitation light at 690 nm [23].

Unlike most cancer therapies which produce apoptotic cell death, NIR-PIT is unique in causing “immunogenic cell death (ICD)” [24], [25]. ICD is a type of cell death in which the adaptive immune system responds to the onslaught of cell-associated antigens released from damaged cancer cells [26], [27]. Apoptotic cell death does not activate the adaptive immune system [28]. ICD is initiated by the release of danger signals, such as calreticulin (CRT), adenosine triphosphate (ATP), high-mobility group box 1 (HMGB1), heat shock protein (Hsp) 70, and Hsp 90 [29], [30]. These danger signals activate immature dendritic cells (DCs) and stimulate the presentation of tumor-antigens to T cells. Cancer cells treated by NIR-PIT release such death signals as CRT, ATP, and HMGB1. Moreover, the activated DCs engulf cancer-specific antigens released from the ruptured tumor cells thereby converting into mature DCs, which can prime and educate naive T cells to become cancer-specific CD8+ T effector cells [24], [31]. NIR-PIT has been shown to convert some non- or low-immunogenic tumors into immunogenic tumors by utilizing innate immunity to recognize newly released cancer-specific antigens. It is therefore, not surprising that NIR-PIT in combination with immune activation therapies (e.g. immune checkpoint inhibitors) has shown an additive effect and even abscopal effects can be observed in mouse models with intact immune systems [31], [32], [33], [34].

Currently, a global Phase 3 clinical trial using an antibody against epidermal growth factor receptor (EGFR) conjugated to IR700 molecule (Cetuximab-IR700) is being tested in patients with recurrent head and neck cancers [35]. NIR-PIT has been given fast-track recognition by the US Food and Drug Administration (FDA). Moreover, the first EGFR targeted NIR-PIT drug (ASP-1929; Akalux™, Rakten Medical Inc.) and a diode laser system (BioBlade™, Rakten Medical Inc.) was conditionally approved and registered for clinical use by the Pharmaceuticals and Medical Devices Agency in Japan in September 2020.

## 3 SUPR effect

### 3.1 Nanoparticle carriers and EPR effect

Chemotherapy has been a mainstay in the treatment of advanced cancer, especially in the late stages. Chemotherapeutic drugs can kill cancer cells effectively but also damage normal cells causing side effects, such as bone marrow suppression, mucositis, neurotoxicity, nausea, vomiting, and hair loss [36], [37]. Since the molecular weight of conventional chemotherapeutic drugs is typically low (<1000 Da), these drugs are delivered everywhere in the body, and therefore, side effects can involve numerous body systems. The serum half-life of conventional chemotherapeutic drugs in blood is short and the off-target accumulation of them in multiple healthy organs is significant, and these two factors result in often severe side effects [38]. Because of rapid progress in nanotechnology, nanoparticle carriers (NCs) have been developed in the last few decades. These typically measure 50–200 nm in diameter and therefore, do not enter most tumors in large amounts. NCs have the advantage of having large payloads of drug. However, typically leakage into the tumor parenchyma by NCs is very limited because of their prolonged circulation time, NCs accumulate in tumors based on what is known as the enhanced permeability and retention (EPR) effect, which was first reported by Matsumura and Maeda in 1986 and has been the basis for developing NCs for tumor-targeted drug delivery ever since [39], [40]. However, the EPR effect is usually subtle and the effect is not large. It arises from abnormalities in the inherent permeability of tumor blood vessels and absence of lymphatic drainage in tumors. Nanomaterials with sizes up to several hundred nanometers slowly extravasate from tumor blood vessels and are retained in tumor beds, leading to a relatively effective and selective accumulation of NCs in solid tumors. However, the EPR effect is misleadingly prominent in small animal xenograft models and frustratingly minimal in most human tumors [41]. While many preclinical studies showed that NCs were effective for tumor treatment, most have not been successful when tested in human clinical trials [42], [43], [44]. Therefore, most of recent studies have tended to investigate approaches to extend the conventional EPR-based targeting with NCs, which is known as “passive targeting”.

### 3.2 Strategies for improving of delivering nanodrugs into tumors

Unlike passive targeting, active targeting, using targeting ligands such as antibodies, fragments of antibodies and peptides, can be a complementary strategy to enhance nanomedicine tumor accumulation and retention. For example, 90Y-ibritumomab tiuxetan (Zevalin^®^), 131I-tositumomab (Bexxar^®^) and denileukin diftitox (Ontak^®^) have been approved for clinical use by the FDA [45], [46]. Triggered drug release can be another complementary strategy. Drug delivery systems made from materials that are sensitive to an external stimulus (e.g. pH, temperature, ultrasound, electrical and magnetic fields, and specific molecules) are designed to release the payload drug only when it has reached the tumor and encounters a release stimulus [47], [48], [49]. As a result, triggered drug release can treat tumors selectively and efficiently while minimizing nonspecific toxicity. Although active targeting and triggered drug release have obvious appeal as delivery strategies, these targeted agents must still enter tumor sites in sufficient concentrations to be effective. Accordingly, adequate passive targeting is required before both strategies can be effective.

### 3.3 Strategies for improving of the drug delivery to tumor sites

The efficiency of passive targeting depends on the permeability of tumor vasculature, interstitial fluid pressure (IFP), and forces exerted by nonfluid components. Tumor vessels are often enlarged, leaky, and exhibit bidirectional flow. These factors tend to create uneven delivery of drugs within the tumor [50]. IFP is often more than 5–10 mmHg within a tumor compared to near zero in normal non tumor tissue decreasing the pressure gradient between capillaries and the extracellular space and thus, reducing diffusion of nanoparticles [51]. Extracellular matrix (ECM) can also be a transport barrier to drug delivery as it can narrow the vasculature, increase the diffusion distance from vessels to the tumor cells; entrap drugs and create a steric obstruction to diffusion of nanoparticles [51]. Tumor cells and the ECM create a solid stress within tumors [52]. The EPR effect can be improved by altering any of the above conditions. There are three main strategies to modify the tumor environment: (i) increase tumor blood flow, (ii) normalize the vessels themselves, and (iii) reduce transcapillary resistance [53].

### 3.4 SUPR effect

**Figure 2:**
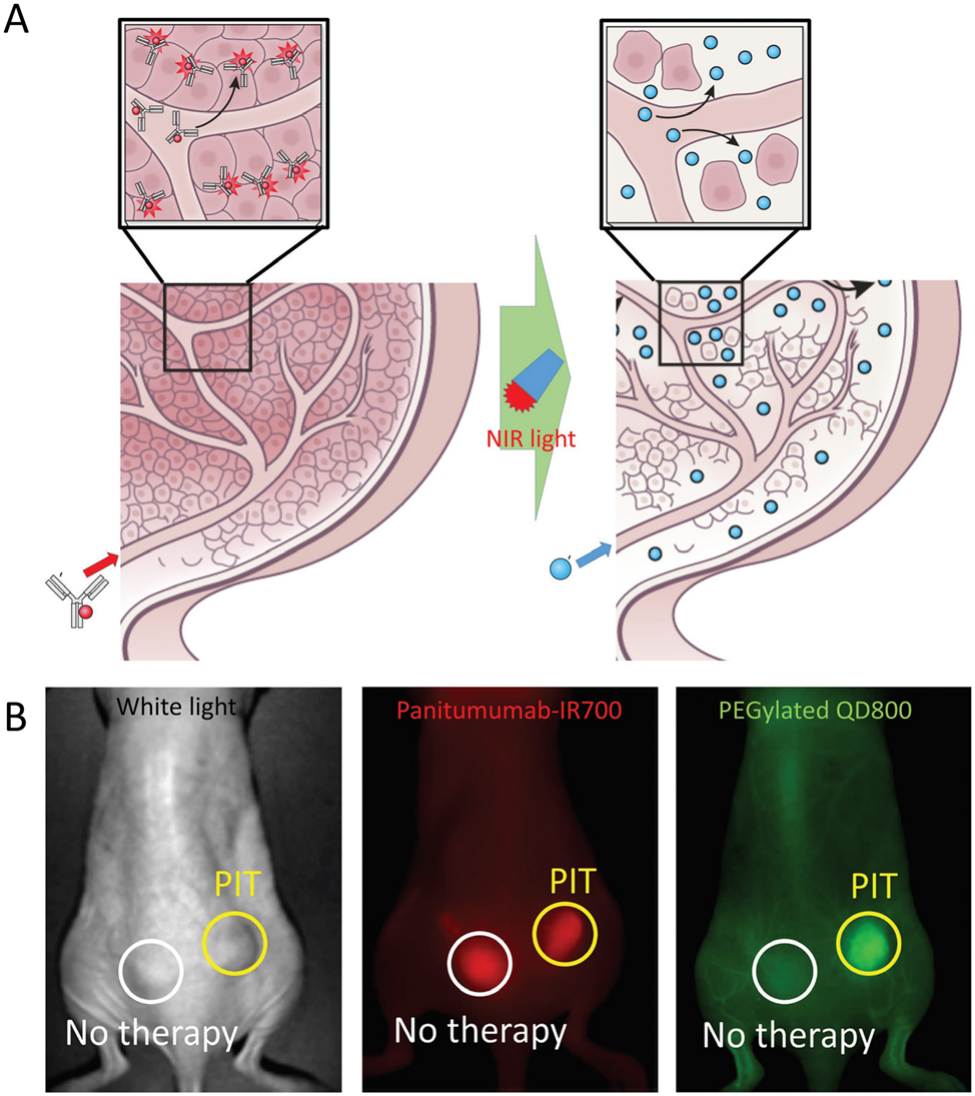
The mechanisms of SUPR effects induced by NIR-PIT. (A) Scheme of SUPR effect induced by NIR-PIT. Many of the initial cell killing occurs in the perivascular layer of tumor cells after NIR-PIT, leading to form a potential space around the tumor vasculature. It increases vascular permeability and decreases interstitial pressures. Then, nanodrug delivery to the remaining tumor can be enhanced. Adapted from Ref. [8]. (B) The increases of PEGylated quantum dot 800 into tumor bed 1 h after NIR-PIT were observed compared to control tumors (up to 24-fold). Adapted from Ref. [8].

NIR-PIT kills cancer cells without destroying surrounding normal cells (e.g. vascular endothelial cells). When APCs arrive at a tumor they leak from the blood vessels and bind to the tumor. By virtue of their proximity to the vessel, the APCs tend to bind to the first cells they encounter, namely perivascular cells. Therefore, the first cells to be killed by NIR-PIT are perivascular tumor cells. The immediate death of perivascular cancer cells creates a potential space between the vessel wall and the remaining tumor which allows nanodrugs to enter the treated tumor beds at dramatically increased concentrations than could be achieved with EPR alone. The drastic increase in permeability and retention in tumor beds following NIR-PIT has been termed the “super-enhanced permeability and retention” (SUPR) effect (Figure 2A) [44, 54, 55]. The SUPR effect allows much higher concentrations of nanodrugs into the tumor, since the initial binding site barrier has been eliminated. Thus, nanodrugs not only accumulate in higher concentrations but can also infiltrate deeper into tumors following NIR-PIT. After NIR-PIT, SUPR effects are always observed permitting the accumulation of many types of nanodrugs of various sizes up to several hundred nanometers in diameter (e.g., monoclonal antibody targeting tumor, nontargeted PEG-coated quantum dots, iron oxide nanoparticles, and dendrimer-based nanosized contrast agents) [54], [56]. Up to 24-fold greater accumulation of untargeted nanoparticles has been measured after NIR-PIT compared to untreated control tumors in which only the conventional EPR effect is present (Figure 2B) [54]. When NIR-PIT was combined with clinically approved nanodrugs such as liposomal daunorubicin (DaunoXome^®^) or albumin-bound paclitaxel (Abraxane^®^), therapeutic effects were significantly enhanced compared to single therapy of either NIR-PIT or nanodrugs [54], [57]. Therefore, the combination of NIR-PIT and nanodrugs could be a promising strategy for increasing the effectiveness of either monotherapy alone.

## 4 NIR-PIT for various cancers

**Figure 3:**
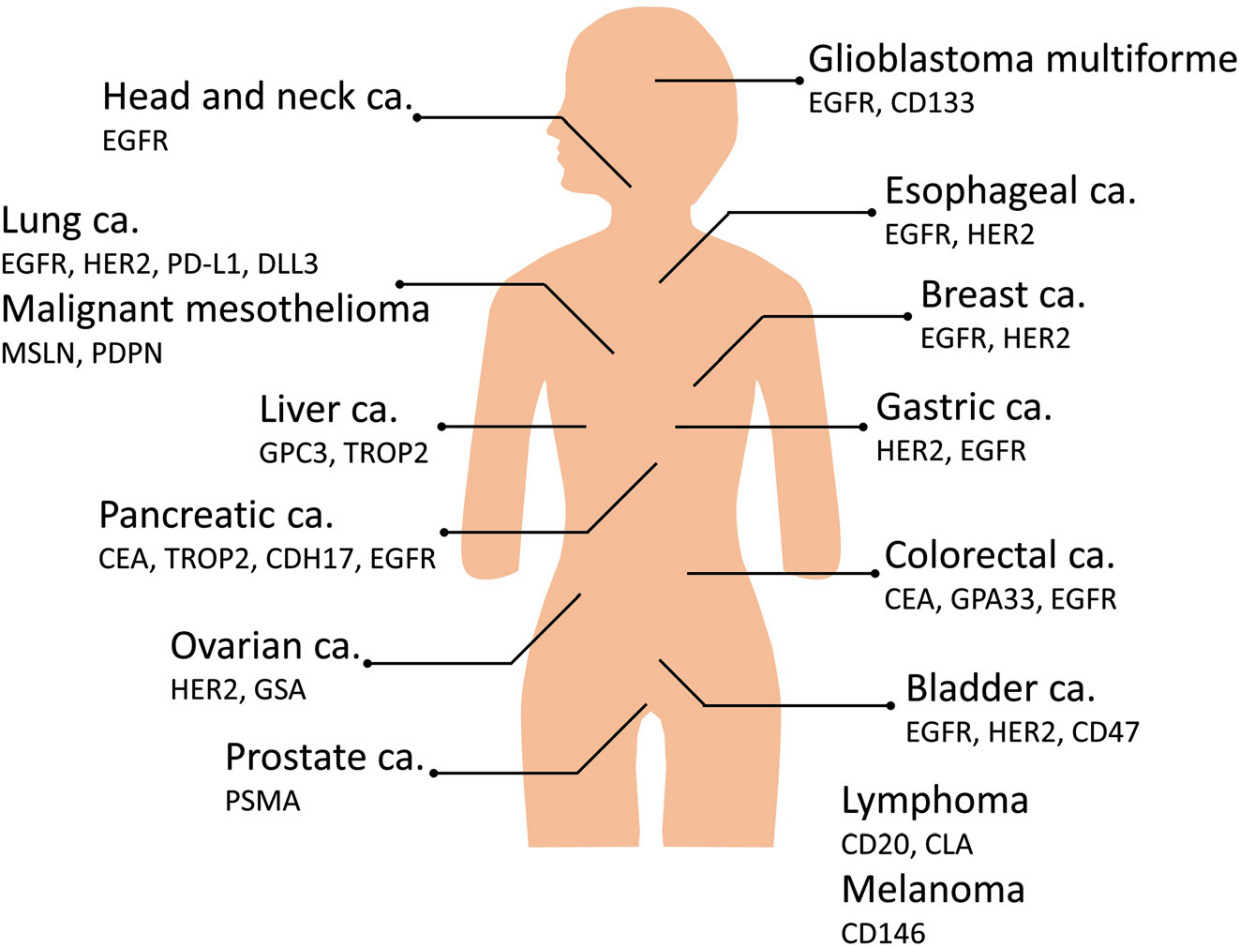
Various cancers and promising targets for NIR-PIT.

NIR-PIT can be applied with any surface marker of cancer or stromal cells provided that an antibody exists to bind to it [8, 58, 59]. In this section, we discuss a range of NIR-PIT applications for various cancers in clinical and preclinical studies (Figure 3).

### 4.1 Head and neck squamous cell carcinoma

Head and neck cancer is the seventh most common cancer with >931,000 new cases worldwide and is seventh the most common cause of cancer death with >467,000 deaths worldwide in 2020 [2]. Head and neck squamous cell carcinoma (HNSCCs) is the most common type of head and neck cancer [60]. Risk factors for HNSCCs are exposure to tobacco-derived carcinogens, excessive alcohol consumption, and human papillomavirus or Epstein–Barr virus infection. Approximately 30–40% of HNSCC patients present at an early stage are curable with surgery or radiotherapy alone. However, multimodality treatment including surgery, radiation, chemotherapy, or immunotherapy is often required for late-stage patients, which constitute more than 60% of patients [61]. The side effects of these combined therapies can damage the delicate structures controlling speech, taste and swallowing leading to debilitating loss of quality of life [62]. Therefore, developing new therapeutic methods which treat the cancer effectively while preserving function is a high priority.

Head and neck cancers are amenable to NIR-PIT because they are often close to the skin or mucosa. Interstitial light fibers can be inserted into deeper tumors. Because head and neck cancers can invade critical vascular structures, care must be taken when treating head and neck cancers with NIR-PIT.

The most clinical experience exists for head and neck NIR-PIT. EGFR is overexpressed in up to 90% of HNSCCs [63]. Cetuximab, which is a chimeric IgG1 monoclonal antibody and a competitive inhibitor of EGFR ligand binding, was approved by the FDA in 2006. Cetuximab-IR700 was the first agent clinically introduced for NIR-PIT. A Phase 1/2 clinical trial of NIR-PIT using cetuximab-IR700 in patients with recurrent HNSCC concluded in 2017 and showed that cetuximab-IR700 NIR-PIT is more effective than current second- and third-line therapies for recurrent HNSCCs [64]. These results prompted the FDA to assign a fast track designation for cetuximab-IR700 in 2018. In September 2020, cetuximab-IR700 received conditional approval from the Japanese Ministry of Health, Labor, and Welfare as a treatment for HNSCC patients. A global phase 3 trial in recurrent HNSCC is currently underway [35]. It is anticipated that NIR-PIT may be used earlier in the disease, including on premalignant lesions in the mouth such as leukoplakia which also express EGFR.

CD44, a cancer stem cell (CSC) marker, is also expressed on HNSCC. Its presence is a negative prognostic indicator and is associated with tumor progression, metastasis and poor prognosis [65]. In mouse homograft models, CD44 targeted NIR-PIT suppressed tumor growth and prolonged survival [32]. Moreover, CD44 targeted NIR-PIT with PD-1 blockade therapy was more effective than single therapies in mouse homograft models including a minimally immunogenic tumor [31], [34].

### 4.2 Glioblastoma multiforme

Glioblastoma multiforme (GBM) is among the most aggressive tumors in adults and carries a dismal 5 year prognosis of only 5.5% [66], [67]. In the United States, an estimated 11,833 patients are diagnosed with GBM per year [68]. The current standard of care for patients younger than age 70 years with newly diagnosed GBM is maximal safe surgical resection, followed by radiation therapy and concomitant temozolomide (TMZ) followed by adjuvant TMZ [66]. For elderly patients performance status can affect treatment decisions and is adjusted according to the ability of the patient to tolerate the intense combination therapy [69]. Even though GBM is well known to extend beyond the visible borders seen on brain MRI, studies of recurrence patterns of GBM after surgery, radiotherapy, and chemotherapy have shown that ∼80–90% of recurrences are within the original treatment field [70], [71], [72], [73], suggesting the efficacy of current therapeutic methods is insufficient. NIR-PIT could be a useful adjuvant to surgery to selectively kill the unresected tumor cells that invade around surgical cavity.

Although NIR light can transmit through the skull it is uncertain whether it is of sufficient intensity to treat GBMs. More likely NIR-PIT will be used with thin fibro optic diffusers through inserted small catheter or as an adjuvant to surgery after the skull is opened. Direct light application to the surgical field could be useful in sterilizing tumor margins. It is possible that wireless LEDs could also be inserted in the surgical cavity to supply light as needed in case of recurrent disease [22].

Several studies have shown EGFR gene amplification in ∼40% of all GBMs [74], [75], [76]. Burley et al. reported that EGFR targeted NIR-PIT showed effectiveness in xenograft models of GBM [77]. Therefore, the most likely first APC to be used in GBM will be cetuximab-IR700.

Therapeutic resistance may also arise from CSCs within GBMs [78], [79]. The neural stem cell marker CD133 has been the CSC marker most associated with GBM and identifies cells with higher rates of self renewal and proliferation and increased differentiation ability [80], [81]. In orthotopic xenograft models, anti-CD133-IR700 conjugates accumulated in brain tumors, suggesting anti-CD133-IR700 can pass through the blood brain barrier. Moreover, CD133 targeted NIR-PIT was highly efficient in both the subcutaneous and orthotopic models [11]. Therefore, EGFR or CSC targeted NIR-PIT is a potential therapy for GBMs.

### 4.3 Esophageal cancer

Esophageal carcinoma is the 11th most common cancer with >604,000 cases worldwide and is the sixth most common cause of cancer death with >544,000 deaths worldwide in 2020 [2]. The two major subtypes of esophageal cancer are esophageal squamous cell carcinoma (ESCC) and esophageal adenocarcinoma (EAC). ESCC represents 90% of all cases of esophageal cancer globally and is dominant in East Asia, East Africa, and South America. EAC is more common in developed countries than in developing countries [82]. Most patients with esophageal cancer need multimodality treatment, including chemotherapy, chemoradiotherapy, and/or surgical resection. Recurrences are particularly difficult to treat.

NIR-PIT would most likely be applied in conjunction with upper gastrointestinal (GI) endoscopy. The operator could identify the tumor location and then apply NIR light to tumors in patients who had previously received the APC. Naturally, treatment of transmural tumors could lead to esophageal perforations so proper patient selection is needed.

Between 71 and 91% of ESCCs and 32–64% of EACs express EGFR and therefore may be potential candidates for EGFR targeted NIR-PIT [83], [84], [85], [86], [87]. Overexpression of human epidermal growth factor 2 (HER2) has been reported in up to 64% of ESCCs and 32% of EACs, respectively [88], [89], [90]. *In vitro* studies, EGFR or HER2 targeted NIR-PIT has been shown to be effective in esophageal carcinoma cell lines [91].

In addition to epithelial growth factor receptors, many studies have emphasized the importance of cancer-associated fibroblasts (CAFs) in esophageal carcinoma [92], [93]. CAF targeted NIR-PIT inhibited tumor progression in co-culture models of ESCCs and CAFs [94], [95]. Therefore, esophageal cancer could be treated with some combination of EGFR, HER2, or CAF targeted NIR-PIT. It is possible in the future that cocktails of APCs could be used to more completely treat particular cancers.

### 4.4 Lung cancer

Lung cancer is the second most common cancer with >2,206,000 new cases worldwide and is the most common cause of cancer death with >1,796,000 deaths worldwide in 2020 [2]. The two major subtypes of lung cancer are non small cell lung cancer (NSCLC) (85% of patients) and small cell lung cancer (SCLC) (15%) [96].

The lungs are a particularly interesting organ to consider treating with NIR-PIT. NIR light transmits through air very well. For lesions near the main bronchi it may be possible to deliver light via a bronchoscope. For deeper lesions a bronchoscope or transcutaneous fiber might be used. It may be possible to treat several lesions simultaneously by applying light to normal lung and having it transmit throughout the lung. Intraoperatively, light could be directed to pleural surfaces or to the lung parenchyma.

EGFR overexpression has been identified in 40–80% of NCSLCs [97]. NIR-PIT with panitumumab (another antibody targeting EGFR)-IR700 conjugates inhibited tumor growth in a transgenic mouse model of spontaneous lung cancer expressing human EGFR [15]. Several monoclonal antibodies (mAbs) against PD-L1 and PD-1 have demonstrated clinical benefit in patients with NSCLC and are collectively known as checkpoint inhibitors [98], [99], [100]. PD-L1 is over expressed in many cancers, and therefore it is a potential target for NIR-PIT. NIR-PIT using avelumab (human antiPD-L1 mAb)-IR700 induced significant therapeutic effects in an NSCLC xenograft model [101]. Thus, PD-L1 targeted NIR-PIT might be useful for NSCLC cancers with high PD-L1 expression. SCLC has a poor prognosis and it is commonly diagnosed at an advanced, unresectable stage [102]. Delta-like protein 3 (DLL3) is a potential therapeutic target molecule for SCLC [103], but rovalpituzumab tesirine, which is the first antibody drug conjugate (ADC) targeting DLL3, was terminated on August 2019 because of failure of both the TAHOE (NCT03061812) and MERU (NCT03033511) clinical trials. The failure of this ADC is not necessarily an indictment of the antibody; however, NIR-PIT targeting DDL3 showed marked antitumor effects [104]. Malignant cells in the pleural fluid or pleural metastases are classified as M1a and stage IV according to TNM Classification of Malignant Tumors 8th edition (UICC 8th edition) and typically surgical resection is excluded [105]. Therefore, therapies that could treat pleural metastases without damage to the adjacent organs might prolong survival. In xenograft models, HER2 targeted NIR-PIT led to significant reduction in pleural dissemination by HER2 expressing NSCLC cells [18]. Furthermore, in mouse models of lung metastasis, HER2 targeted NIR-PIT showed significant reductions in metastasis tumor volume and prolonged survival [16], [17]. These results suggest a potential new therapy for the local control of lung metastases or pleural dissemination which could readily be translated to clinical treatments.

### 4.5 Malignant pleural mesothelioma

Malignant pleural mesothelioma (MPM) is a malignant tumor that originates from mesothelial cells in the pleura and peritoneum and has an extremely poor prognosis, with a median survival of 8–14 months [106]. It is often debulked with extrapleural pneumonectomies but recurrence is common.

NIR-PIT could be considered at the time of the debulking procedure. At that time the mesothelioma would be exposed and NIR light could be directly applied to the tumor surface. It may also be possible to deliver light to the pleura broncho-scopically in some cases.

Podoplanin (PDPN) is a type I transmembrane glycoprotein that is expressed in lymphatic endothelial cells, type I alveolar epithelial cells, and podocytes of the glomeruli. PDPN is a specific pathological diagnostic marker to distinguish lymphatic vessels from blood vessels but it is also expressed in MPM [107], [108], [109]. PDPN targeted NIR-PIT in MPM models suppressed tumor progression [110]. Another MSM marker, mesothelin (MSLN) is a cell surface glycoprotein that is a target for antibody-based therapies [111], [112]. MSLN targeted NIR-PIT has been shown to be effective in mouse xenograft models [113]. These findings suggest that PDPN and/or MSLN targeted NIR-PIT might be a potential alternative treatment of MPM.

### 4.6 Breast cancer

Female breast cancer is the most common cancer with >2,260,000 new cases worldwide and is the fifth most common cause of cancer death with >684,000 deaths worldwide in 2020 [2]. Breast cancer is classified into three major subtypes based on the presence or absence of molecular markers for two hormone receptors (HR) and HER2. HER2, which is a member of the epidermal growth factor receptor family, regulates cell proliferation, differentiation, and apoptosis through signal transduction. Tumors are classified by their HR and HER2 status: HR+/HER2− (70% of patients), HR±/HER2+ (15–20%), and “triple-negative” (HR−/HER2−; 10–15%) [114]. Breast cancer is treated by multimodal therapy. Locoregional therapies include surgery and radiation therapy. Systemic therapies include hormone therapy for HR+ patients, chemotherapy, HER2 targeted therapy for HER2+ patients, bone-modifying agents, poly (ADP-ribose) polymerase (PARP) inhibitors for BRCA-mutated cancer and immunotherapy [114], [115], [116], [117].

NIR-PIT could be used in a variety of ways in breast cancer. For localized disease it could be a method of treating the cancer with interstitial fibers. For local recurrences or recurrences in the chest wall a similar strategy could be used. For lung metastases it might be possible to illuminate the lungs to deliver light.

Trastuzumab, an anti-HER2 mAb has been used as an NIR-PIT agent in xenograft models of breast cancer [7]. Cells that are unresponsive to HER2 targeted NIR-PIT are often shown to be HER2 targeted NIR-PIT-responsive after viral transduction of the HER2-extracellular domain [118]. For triple-negative breast tumors, no targeted therapy is currently available. However, EGFR expression has been reported in 50–89% of cases [119], [120]. Therefore, EGFR targeted NIR-PIT could be used in some cases of triple-negative breast cancer. In xenograft models with two different cell lines established from triple-negative breast cancers, cetuximab-IR700 NIR-PIT suppressed tumor growth and prolonged survival [121].

### 4.7 Gastric cancer

Gastric cancer is the sixth most common cancer with >1,089,000 new cases worldwide and is the fourth most common cause of cancer death with >768,000 deaths worldwide in 2020 [2]. *Helicobacter pylori* infection has been implicated in more than 93% of gastric cancer patients [122]. In the trastuzumab for gastric cancer trial (ToGA trial), HER2 positivity was 22.1% [89]. The FDA approved trastuzumab (anti-HER2 mAb) in combination with chemotherapy as a standard treatment for patients with HER2+ advanced gastric or gastro-esophageal junction cancer in 2010 [123]. In mouse models of peritoneal carcinomatosis or a flank tumor, HER2 targeted NIR-PIT showed significant reductions in tumor volume [124]. The combination therapy of HER2 targeted NIR-PIT (using Trastuzumab-IR700) and conventional chemotherapy of 5-FU rapidly induced significant tumor inhibition [125]. Currently, anti-HER2 antibodies recognizing different epitopes of HER2 have been developed, such as pertuzumab which could also be used for NIR-PIT. NIR-PIT with trastuzumab-IR700 and pertuzumab-IR700 conjugates showed stronger antitumor effects than either antibody conjugate alone [126]. Less than 6% of the normal gastric tissues demonstrated EGFR expression, whereas EGFR was expressed in 41.8–57.7% of gastric cancers by immunohistochemistry (IHC) analysis [127], [128], [129]. Therefore, EGFR targeted NIR-PIT, perhaps in combination with HER2 targeted NIR-PIT might be a suitable treatment for some patients with gastric cancer.

### 4.8 Colorectal cancer

Colorectal cancer is the third most common cancer with >1,880,000 new cases worldwide and is the second most common cause of cancer death with >915,000 deaths worldwide in 2020. The incidence rate of colorectal cancer is higher in developed countries than in developing countries [2]. With economic growth in developing countries, it is estimated that the incidence of colorectal cancer could increase to 2.5 million new cases by 2035 [130]. Carcinoembryonic antigen (CEA) is preferentially expressed in colon cancer cells compared to normal colon cells [131], [132]. Moreover, overexpression of CEA in tumor tissue is associated with a negative prognostic sign [133], [134]. CEA targeted NIR-PIT inhibited tumor growth in a CEA-expressing mouse xenograft model [135], [136].

Another potential target for NIR-PIT in colon cancer is the glycoprotein A33 antigen (GPA33) which is highly expressed in over 95% of human colorectal cancers and exhibits limited expression in normal intestinal epithelium [137]. GPA33 targeted NIR-PIT showed significant efficacy in xenograft models [138]. EGFR overexpression has been observed in 43.9–97% of colorectal cancer patients based on IHC analysis [139], [140], [141], [142]. Some of the anti-EGFR mAbs including cetuximab and panitumumab have been approved by FDA as first-line treatments of colorectal cancer [143]. Thus, EGFR targeted NIR-PIT could be utilized as adjuvant therapy in conjunction with surgery or laparoscopy.

### 4.9 Liver cancer

Liver cancer is the eighth most common with >905,000 new cases worldwide and is the third most common cause of cancer death with >830,000 deaths worldwide in 2020 [2]. Hepatocellular carcinoma (HCC) is the most common type of primary hepatic malignancy. HCC is caused by chronic viral hepatitis which results in cirrhosis and tumor formation. Treatments include surgery, radiation, chemotherapy, immunotherapy, and liver transplantation. In general, mortality rates are high for HCC.

Since HCC is usually found within the liver parenchyma it would have to be approached with catheters or fiber optic needles. The liver itself is relatively poor at light transmission so it is important that the fiber optic needles would be placed within the tumor itself. In the future it may be possible to leave in place wireless LEDs to provide light in the event of recurrence after NIR-PIT.

Glypican-3 (GPC3) is highly expressed in HCC but not in normal tissue, and therefore, is a target-candidate for NIR-PIT [144]. GPC3 targeted NIR-PIT inhibited tumor growth compared to untreated controls in a xenograft model of HCC [145]. Moreover, the combination of GPC3 targeted NIR-PIT and nanoparticle albumin-bound paclitaxel enhanced the therapeutic effect compared to either alone [57]. The post NIR-PIT SUPR effect enabled more drug delivery to the tumor. Cholangiocarcinoma (CCA) is the second most common liver cancer after HCC accounting for ∼15% of all primary liver cancers and ∼3% of all gastrointestinal cancers worldwide [146]. It is difficult to deliver NIR light into the bile duct for CCA from outside the body. However, newly developed catheter-based devices containing LEDs could be used to deliver light for NIR-PIT resulting in tumor suppression in xenograft models [147]. Tumor-associated calcium signal transducer 2 (TROP2) is overexpressed in many epithelial cancers including CCA [148] and TROP2 corelates with a poor prognosis in various cancers [149]. TROP2 targeted NIR-PIT inhibited tumor growth in CCA xenograft model [150].

### 4.10 Pancreatic cancer

Pancreatic cancer is the 14th most common cancer with >495,000 new cases worldwide and the eighth most common cause of cancer death with >466,000 deaths worldwide in 2020 [2]. Pancreatic cancer is aggressive and often presents late because symptoms are nonspecific or minimal early in the disease. The pancreatic cancer cell is particularly aggressive and the tumor recurrence rate after radical surgical resection is 80% even though surgical techniques and adjuvant treatments have improved in the last decades [151].

The pancreas primary is difficult to approach with light. It is possible, depending on the tumor location in the pancreas, that endoscopic-ultrasound-light delivery could provide light to the tumor. More realistically, NIR-PIT could be performed as an adjuvant to open or laparoscopic surgery. Given the high rate of recurrence it may be especially beneficial to have a wide field of NIR-PIT in pancreatic cancer.

Among the targets potentially suitable for NIR-PIT targeting is anti-CEA mAb-IR700 which demonstrated a good response in an orthotopic xenograft model [152]. Cadherin-17 (CDH17) is highly expressed on gastrointestinal cancer cells. CDH17 targeted NIR-PIT inhibited tumor growth in a xenograft model which used a pancreatic cancer cell line [153]. TROP2 is also overexpressed in pancreatic cancer [148]. TROP2 targeted NIR-PIT inhibited tumor growth in pancreatic cancer xenograft models [150]. Moreover, CEA targeted NIR-PIT following surgery reduced recurrence by eliminating remaining cancer cells [154], [155]. EGFR expression was observed in 62–69% of pancreatic cancer patients with IHC analysis [156], [157], [158]. Thus, some combination of antibody conjugates engaging CEA, CDH17, TROP2, and EGFR might be successful in pancreatic cancer.

### 4.11 Ovarian cancer

Ovarian cancer is the 19th most common cancer with >313,000 new cases worldwide and the 15th most common cause of cancer death with >207,000 deaths worldwide in 2020 [2]. Since ovarian cancer is a highly metastatic disease, a minority (15%) of patients is diagnosed with localized tumor (stage I) however, when early diagnosis can be made the 5-year survival is 92%. However, the majority of cases present late with disseminated intra abdominal disease (stages III–IV) with a 5-year survival of 25% [159], [160]. Standard therapy of ovarian cancer is a combination of chemotherapy and surgery. Cytoreductive surgery is initially employed to remove all macroscopic disease (R0 resection). The success of the R0 resection is a prognostic indicator [161], [162]. When the disease is considered to be R1 (<1 cm), all visible disease is removed but viable microscopic cancer cells are assumed to remain at the surgical margin and recurrence is common [160], [163]. Therefore, the development of new therapies for treating residual disease in the peritoneum after cytoreductive surgery is needed.

A typical pattern of recurrence is in the peritoneum where direct light exposure is possible during open or laparoscopic procedures. Lymph node metastases present a greater problem and may require direct light exposure during surgery. While NIR-PIT might not be curative in all cases it could be very useful in killing recurrent peritoneal disease thus staving off the primary symptom of recurrent ovarian cancer, malignant ascites.

HER2 targeted NIR-PIT showed significant tumor suppression in subcutaneous tumor models but also in disseminated peritoneal models using HER2 expressing ovarian cancer cell lines [164]. Galactosyl serum albumin (GSA) binds to beta-d-galactose receptors, which is overexpressed on the surface of many ovarian tumors [165]. GSA targeted NIR-PIT specifically killed ovarian cancer cells (SHIN3) *in vitro* and suppressed tumor growth in a peritoneal disseminated model [166].

### 4.12 Bladder cancer

Bladder cancer is the 13th most common cancer with >573,000 new cases and is the 14th most common cause of cancer death with >212,000 deaths worldwide in 2020 [2]. Evaluation of bladder cancer patients is performed using cystoscopy with a flexible scope [167]. EGFR and HER2 were detected in 72.2 and 44.5% of bladder cancers, respectively [168]. Therefore, these receptors could be targets for NIR-PIT in bladder cancer. EGFR targeted NIR-PIT caused cell death in human bladder tumor cell lines *in vitro* and inhibited tumor growth in bladder tumor xenograft models [169], [170]. Moreover, combined EGFR and HER2 targeted NIR-PIT inhibited tumor growth significantly in a xenograft bladder tumor model [171]. CD47, one of the “don’t eat me” signals for macrophages, is also highly expressed (80%) in bladder cancer tumors, but is not expressed on normal luminal urothelium [172]. Kiss et al. reported that CD47 targeted NIR-PIT killed human bladder tumor cell lines and patient derived bladder tumor cells *in vivo*, and suppressed tumor growth in xenograft models [173].

### 4.13 Prostate cancer

Prostate cancer is the fourth most common cancer with >1,414,000 new cases and is the ninth most common cause of cancer death with >375,000 deaths worldwide in 2020 [2]. Prostate cancer is treated by surgery, radiation or active surveillance in the case of low-grade cancers. Because of the high morbidity of prostate cancer treatments (urinary incontinence and erectile dysfunction) alternative therapies have been proposed. Focal therapies consist of ablative methods that physically destroy prostate cancers but also tend to undertreat infiltrative disease while damaging normal prostate tissue.

Prostate cancer NIR-PIT could be relatively straightforward as the prostate is commonly biopsied using MRI-Ultrasound fusion imaging. Instead of biopsy needles, fiber optic needles could be introduced into the prostate enabling the killing of cancer cells while sparing critical structures like the urethra, sphincters, and pelvic nerves.

Prostate-specific membrane antigen (PSMA) is overexpressed significantly in prostatic cancer cells and the expression level of PSMA is associated with the stage and grade of the prostate cancer but the expression is low in normal tissues [174]. Thus, PSMA is a reasonable target for molecular therapy. PSMA targeted NIR-PIT eliminated prostate tumor cells significantly *in vivo* and suppressed tumor progression and prolonged survival in xenograft models [175]. Moreover, PSMA targeted NIR-PIT using anti-PSMA diabody (Db) or anti-PSMA minibody (Mb), which are small and bivalent antibody fragments of anti-PSMA-IgG, showed PSMA+ cell death *in vitro* and suppressed tumor growth in a xenograft model [176]. A major advantage of PSMA targeted NIR-PIT is that suitable patients could be determined using PSMA PET scans prior to the procedure [177]. This would localize the tumor accurately and allow placement of fiber optic catheters. Following NIR-PIT the same PSMA PET scan could be used to determine the success of the procedure and whether additional treatments might be needed.

### 4.14 Lymphoma

Lymphoma is the ninth most common cancer with >627,000 new cases worldwide and is the 12th most common cause of cancer death with >283,000 deaths worldwide in 2020 [2]. Traditionally, lymphoma is divided into Hodgkin’s lymphoma (approx. 13% of all lymphomas) and non-Hodgkin’s lymphoma [2]. The majority of lymphomas are B cell origin [178]. B-cell lymphomas often express B-cell markers, such as CD19 and CD20, which can bind specific mAbs [179]. CD20 targeted NIR-PIT using rituximab-IR700 conjugates showed efficacy in xenograft models of B-cell lymphoma [180]. Furthermore, the therapeutic effect of CD20 targeted NIR-PIT was more effective than that of radioimmunotherapy in a xenograft model of aggressive B-cell lymphoma [181]. Mycosis fungoides (MF) is a rare cancer however; it is the most common subtype of cutaneous T-cell lymphoma [182]. It is reported that MF cells express cutaneous lymphocyte antigen (CLA) [183]. CLA targeted NIR-PIT specifically killed MF cell line *in vitro* [184]. Thus, NIR-PIT could treat the locoregional lymphomas including skin-based lymphoma which would be highly amenable to direct NIR light exposure.

### 4.15 Melanoma

Melanoma is the most aggressive and the lethal form of skin cancer. Melanoma is treated by multimodal therapies, such as surgical resection, chemotherapy, PDT, immunotherapy, and targeted therapy using small molecule inhibitors or antibodies [185].

Because early melanoma is usually present on the skin surface it is relatively amenable to direct NIR light exposure, however, deeper nodal involvement might require interstitial placement of catheters/fiber optics in order to delivery light to all facets of the tumor.

CD146 has been identified as a melanoma cell adhesion molecule. CD146 is overexpressed in 70% of primary melanomas and 90% of lymph node metastases [186]. Wei et al. reported that CD146 targeted NIR-PIT inhibited tumor growth in CD146-positive melanoma xenograft model [187]. Thus, CD146 targeted NIR-PIT could be a potential method of treating melanomas without highly disfiguring surgery.

### 4.16 Bone metastases

Bone is the third most common site of metastases in cancer patients [188]. It has been assumed that NIR-PIT for bone metastases would have no effect because light cannot penetrate bone. However, ex vivo experiments showed that NIR-PIT not only penetrates bone but can kill tumor cells located behind bone. Moreover, tumor viability was reduced by NIR-PIT [189]. Hence, in spite of decreasing of light transmittance, NIR-PIT nonetheless is able to treat cancers within bone.

## 5 Conclusion

NIR-PIT is a new cancer therapy with broad applications. It has an immediate effect on the tumor neovasculature which results in the SUPR effect which enables nanodrugs to penetrate into the treated tumor at far higher concentrations than are normally possible. It also profoundly activates the immune system both locally and, in some cases, systemically. NIR-PIT kills cancers in a highly specific manner and therefore, could be used in a variety of cancers. Each cancer requires one or more specific antibodies that bind the tumor and can be conjugated with IR700 to become an NIR-PIT agent. Using cocktails of mAb-IR700 conjugates that are injected intravenously and various methods of delivering light, a wide range of tumors could be treated with minimal side effects. Recently, EGFR targeted NIR-PIT was conditionally approved in Japan and a phase 3 clinical trial is ongoing. Thus, NIR-PIT has great potential to become a widely applicable cancer therapy in the near future.

## Supplementary Material

Supplementary Material
