# Supplementary material for: Near infrared photoimmunotherapy of cancer; possible clinical applications
Source: Nanophotonics. 2021 May 7;10(12):3135–51. doi: 10.1515/nanoph-2021-0119 (PMC9646249; doi:10.1515/nanoph-2021-0119)
Supplement: Supplementary file 1 — Supplementary Material [file j_nanoph-2021-0119_suppl.docx]

**Near infrared photoimmunotherapy of cancer; possible clinical applications**

Hiroaki Wakiyama^1^, Takuya Kato^1^, Aki Furusawa^1^, Peter L. Choyke^1^, Hisataka Kobayashi^1^

^1^Molecular Imaging Branch, Center for Cancer Research, National Cancer Institute, NIH, Bethesda, MD, 20892, USA

**Funding:** This work was supported by the Intramural Research Program of the National Institutes of Health, National Cancer Institute, Center for Cancer Research [grant numbers ZIA BC 011513

**Corresponding author:**

Hisataka Kobayashi, M.D., Ph.D.

Molecular Imaging Branch, Center for Cancer Research, National Cancer Institute, NIH, 10 Center Drive, Bethesda, MD, 20892, USA

Tel: 240-858-3069; Fax: 240-541-4527; E-mail: kobayash@mail.nih.gov

**Disclosure of Potential Conflicts of Interest:** No potential conflicts of interest were disclosed.

**Keywords:** cancer; near-infrared photoimmunotherapy (NIR-PIT); anti-cancer host immunity; super-enhanced permeability and retention (SUPR) effects; immunogenic cell death.

**Abstract:** Near-infrared photoimmunotherapy (NIR-PIT) is a new cancer treatment that uses an antibody photo-absorber conjugate (APC) composed of a targeting monoclonal antibody (mAb) conjugated with a photoactivatable phthalocyanine-derivative dye, IRDye700DX (IR700). APCs injected into the body can bind to cancer cells where they are activated by local exposure to NIR light typically delivered by a NIR laser. NIR-light alters the APC chemical conformation inducing damage to cancer cell membranes, resulting in necrotic cell death within minutes of light exposure. NIR-PIT selectivity kills cancer cells by immunogenic cell death (ICD) with minimal damage to adjacent normal cells thus, leading to rapid recovery by the patient. Moreover, since NIR-PIT induces ICD only on cancer cells, NIR-PIT initiates and activates anti-tumor host immunity that could be further enhanced when combined with immune checkpoint inhibition. NIR-PIT induces dramatic changes in the tumor vascularity causing the super-enhanced permeability and retention (SUPR) effect that dramatically enhances nanodrug delivery to the tumor bed. Currently, a worldwide Phase 3 study of NIR-PIT for recurrent or inoperable head and neck cancer patients is underway. In September 2020, the first APC and accompanying laser system were conditionally approved for clinical use in Japan. In this review, we introduce NIR-PIT and the SUPR effect and summarize possible applications of NIR-PIT in a variety of cancers.

**1. Introduction**

Cancer is the second leading cause of death globally [1]. It is estimated that 19.3 million new cancer cases and almost 10.0 million cancer deaths occurred in 2020. Moreover, cancer incidence and mortality is rapidly rising worldwide, reflecting aging and environmental exposures [2]. Three major cancer therapies; surgery, radiation therapy, and chemotherapy, have been the mainstay of cancer treatment for many decades. Each method can reduce cancer burden, however, each treatment also causes severe collateral damage to normal cells including immune cells and stem cells contributing to disease recurrence and delayed healing, and resulting in significant consequences for quality of life. In the last decade, improved cancer immunotherapies have dramatically altered the therapeutic landscape [3]. However, the effectiveness of immunotherapy depends on altering the careful balance of effector T cells and immune suppressor cells [4]. Although it can produce spectacular results, the overall response rate of immunotherapy remains relatively low, mostly because of the absence of T cell infiltration in tumors [5]. Meanwhile, immunotherapy-related side effects, termed immune-related adverse events (irAEs), have been widely reported and often mimic autoimmune disease. It has been reported that irAEs were observed in up to 90% of patients treated with an anti-CTLA-4 drug and 70% of those treated with a PD-1/PD-L1 inhibitor, two common checkpoint inhibitors [6]. Thus, despite advances, no cancer treatment is capable of selectively killing cancer cells while activating the local host immune response. Near-infrared photoimmunotherapy (NIR-PIT) is proposed as a method to overcome these challenges.

In this review, we first provide an overview of NIR-PIT. Then, we describe how NIR-PIT can enhance nano-drug delivery based on the super-enhanced permeability and retention (SUPR) effect. Finally, we discuss possible clinical applications of NIR-PIT and SUPR to cancers arising in various organs.

**2. NIR-PIT**

NIR-PIT is a newly developed cancer treatment that employs an antibody conjugated with the NIR photon-absorbing silicon phthalocyanine dye, IRDye700DX (IR700) [7, 8]. This antibody-photon absorber conjugate (APC) is injected intravenously where it binds to specific cancer cells expressing the appropriate antigen on the cell membrane. NIR light (∼690 nm) is then directed to the tumor site activating the APC to induce cell killing [7, 9-11]. Recently the mechanism of cytotoxicity of NIR-PIT has been explained [12]. Immediately after NIR light exposure, axial ligands of the IR700 molecule, which are responsible for its hydrophilicity, are dissociated from the main molecule causing the APC to change from a highly hydrophilic to a highly hydrophobic compound (Figure 1A). This change in chemical properties of the APC promotes aggregation leading to damage and rupture of the cellular membrane. The cell membrane is progressively weakened, micro-perforations form and ultimately blebbing and bursting occurs, resulting in necrotic cell death (Figure 1B). Damage to the cellular membrane during NIR-PIT can be observed with such techniques as three-dimensional low-coherent quantitative phase microscopy or dual-view inverted selective plane illumination microscopy [13]. Movies of cells undergoing cell death during NIR-PIT reveal a rapid swelling of the cell, blebbing and rupture with release of the intracellular contents into the extracellular space. This mechanism of cell death clearly distinguishes NIR-PIT from conventional photodynamic therapy (PDT), which relies on the production of reactive oxygen species to cause non-selective damage to adjacent normal tissue.

In theory, NIR-PIT is most suited to treating superficial tumors because NIR light can penetrate only approximately 2 cm from the tissue surface [14]. In special circumstances, such as treating tumors in the lung and pleural cavity, NIR light can be transmitted much further through the air in the lungs [15-18]. However, in more solid tissues NIR-light is rapidly attenuated and thus, the light source must be placed into or nearby tumors [19]. This can be accomplished by using flexible, cylindrical, fiberoptic, interstitial light diffusers that are inserted into the treatment site. Using interstitial light diffusers practically any tumor site is amenable to NIR-PIT whether inserted via needle, catheter or endoscope [20, 21]. Furthermore, implanted wireless NIR light emitting diode (LED) sources can be used to generate light repeatedly in a remote tumor site [22]. Since the IR700 dye is both a therapeutic and a diagnostic, fluorescence imaging can be used to detect sites of tumor to which the APC is bound and direct therapeutic doses of light to those fluorescing regions. As the light photo-bleaches the IR700 dye, there is a decrease in fluorescence reaching a minimum plateau after the dye is completely photobleached. It is reported that the therapeutic effect correlates with the photo bleaching extent [12]. A commercially available camera, originally designed to image indocyanine green (ICG), which typically operates at wavelengths of 830nm, can be repurposed to detect the low level fluorescence arising from IR700 during NIR-PIT because of the high intensity of the excitation light and the emission spectrum of IR700 which extends beyond 830nm. This enables NIR-PIT to be monitored in real time at wavelengths far from the intense laser excitation light at 690nm [23].

Unlike most cancer therapies which produce apoptotic cell death, NIR-PIT is unique in causing “immunogenic cell death (ICD)” [24, 25]. ICD is a type of cell death in which the adaptive immune system responds to the onslaught of cell-associated antigens released from damaged cancer cells [26, 27]. Apoptotic cell death does not activate the adaptive immune system [28]. ICD is initiated by the release of danger signals, such as calreticulin (CRT), adenosine triphosphate (ATP), high-mobility group box 1 (HMGB1), heat shock protein (Hsp) 70, and Hsp90 [29, 30]. These danger signals activate immature dendritic cells (DCs) and stimulate the presentation of tumor-antigens to T-cells. Cancer cells treated by NIR-PIT release such death signals as CRT, ATP and HMGB1. Moreover, the activated DCs engulf cancer-specific antigens released from the ruptured tumor cells thereby converting into mature DCs, which can prime and educate naive T cells to become cancer-specific CD8+ T effector cells [24, 31]. NIR-PIT has been shown to convert some non- or low-immunogenic tumors into immunogenic tumors by utilizing innate immunity to recognize newly released cancer-specific antigens. It is therefore, not surprising that NIR-PIT in combination with immune activation therapies (e.g. immune checkpoint inhibitors) has shown an additive effect and even abscopal effects can be observed in mouse models with intact immune systems [31-34].

Currently, a global Phase 3 clinical trial using an antibody against epidermal growth factor receptor (EGFR) conjugated to IR700 molecule (Cetuximab-IR700) is being tested in patients with recurrent head and neck cancers [35]. NIR-PIT has been given fast-track recognition by the US Food and Drug Administration (FDA). Moreover, the first EGFR-targeted NIR-PIT drug (ASP-1929; Akalux^TM^, Rakten Medical Inc.) and a diode laser system (BioBlade^TM^, Rakten Medical Inc.) was conditionally approved and registered for clinical use by the Pharmaceuticals and Medical Devices Agency in Japan in September 2020.

**3. SUPR effect**

**3.1 Nanoparticle carriers and EPR effect**

Chemotherapy has been a mainstay in the treatment of advanced cancer, especially in the late stages. Chemotherapeutic drugs can kill cancer cells effectively but also damage normal cells causing side effects, such as bone marrow suppression, mucositis, neurotoxicity, nausea, vomiting and hair loss [36, 37]. Since the molecular weight of conventional chemotherapeutic drugs is typically low (< 1000 Da), these drugs are delivered everywhere in the body, and therefore, side effects can involve numerous body systems. The serum half-life of conventional chemotherapeutic drugs in blood is short and the off-target accumulation of them in multiple healthy organs is significant, and these two factors result in often severe side effects [38]. Because of rapid progress in nanotechnology, nanoparticle carriers (NCs) have been developed in the last few decades. These typically measure 50-200nm in diameter and therefore, do not enter most tumors in large amounts. NCs have the advantage of having large payloads of drug. However, typically leakage into the tumor parenchyma by NCs is very limited Because of their prolonged circulation time, NCs accumulate in tumors based on what is known as the Enhanced Permeability and Retention (EPR) effect, which was first reported by Matsumura and Maeda in 1986 and has been the basis for developing NCs for tumor-targeted drug delivery ever since [39, 40]. However, the EPR effect is usually subtle and the effect is not large. It arises from abnormalities in the inherent permeability of tumor blood vessels and absence of lymphatic drainage in tumors. Nanomaterials with sizes up to several hundred nanometers slowly extravasate from tumor blood vessels and are retained in tumor beds, leading to a relatively effective and selective accumulation of NCs in solid tumors. However, the EPR effect is misleadingly prominent in small animal xenograft models and frustratingly minimal in most human tumors [41]. While many pre-clinical studies showed that NCs were effective for tumor treatment, most have not been successful when tested in human clinical trials [42-44]. Therefore, most of recent studies have tended to investigate approaches to extend the conventional EPR-based targeting with NCs, which is known as “passive targeting”.

**3.2 Strategies for improving of delivering nanodrugs into tumors**

Unlike passive targeting, active targeting, using targeting ligands such as antibodies, fragments of antibodies and peptides, can be a complementary strategy to enhance nanomedicine tumor accumulation and retention. For example, 90Y-ibritumomab tiuxetan (Zevalin®), 131I-tositumomab (Bexxar®) and denileukin diftitox (Ontak®) have been approved for clinical use by the FDA [45, 46]. Triggered drug release can be another complementary strategy. Drug delivery systems made from materials that are sensitive to an external stimulus (e.g. pH, temperature, ultrasound, electrical and magnetic fields, and specific molecules) are designed to release the payload drug only when it has reached the tumor and encounters a release stimulus [47-49]. As a result, triggered drug release can treat tumors selectively and efficiently while minimizing nonspecific toxicity. Although active targeting and triggered drug release have obvious appeal as delivery strategies, these targeted agents must still enter tumor sites in sufficient concentrations to be effective. Accordingly, adequate passive targeting is required before both strategies can be effective.

**3.3 Strategies for improving of the drug delivery to tumor sites**

The efficiency of passive targeting depends on the permeability of tumor vasculature, interstitial fluid pressure (IFP), and forces exerted by nonfluid components. Tumor vessels are often enlarged, leaky and exhibit bidirectional flow. These factors tend to create uneven delivery of drugs within the tumor [50]. IFP is often more than 5–10 mmHg within a tumor compared to near zero in normal non-tumor tissue decreasing the pressure gradient between capillaries and the extracellular space and thus, reducing diffusion of nanoparticles [51]. Extracellular matrix (ECM), can also be a transport barrier to drug delivery as it can narrow the vasculature, increase the diffusion distance from vessels to the tumor cells; entrap drugs and create a steric obstruction to diffusion of nanoparticles [51]. Tumor cells and the ECM create a solid stress within tumors [52]. The EPR effect can be improved by altering any of the above conditions. There are three main strategies to modify the tumor environment: (i) increase tumor blood flow, (ii) normalize the vessels themselves, and (iii) reduce transcapillary resistance [53].

**3.4 SUPR effect**

NIR-PIT kills cancer cells without destroying surrounding normal cells (e.g. vascular endothelial cells). When APCs arrive at a tumor they leak from the blood vessels and bind to the tumor. By virtue of their proximity to the vessel, the APCs tend to bind to the first cells they encounter, namely perivascular cells. Therefore, the first cells to be killed by NIR-PIT are perivascular tumor cells. The immediate death of perivascular cancer cells creates a potential space between the vessel wall and the remaining tumor which allows nanodrugs to enter the treated tumor beds at dramatically increased concentrations than could be achieved with EPR alone. The drastic increase in permeability and retention in tumor beds following NIR-PIT has been termed the “super-enhanced permeability and retention” (SUPR) effect (Figure 2A) [44, 54, 55]. The SUPR effect allows much higher concentrations of nanodrugs into the tumor, since the initial binding site barrier has been eliminated. Thus, nanodrugs not only accumulate in higher concentrations but can also infiltrate deeper into tumors following NIR-PIT. After NIR-PIT, SUPR effects are always observed permitting the accumulation of many types of nanodrugs of various sizes up to several hundred nanometers in diameter (e.g., monoclonal antibody targeting tumor, nontargeted PEG-coated quantum dots, iron oxide nanoparticles, and dendrimer-based nanosized contrast agents) [54, 56]. Up to 24-fold greater accumulation of untargeted nano-particles has been measured after NIR-PIT compared to untreated control tumors in which only the conventional EPR effect is present (Figure 2B) [54]. When NIR-PIT was combined with clinically approved nanodrugs such as liposomal daunorubicin (DaunoXome®) or albumin-bound paclitaxel (Abraxane®), therapeutic effects were significantly enhanced compared to single therapy of either NIR-PIT or nanodrugs [54, 57]. Therefore, the combination of NIR-PIT and nanodrugs could be a promising strategy for increasing the effectiveness of either monotherapy alone.

**4. NIR-PIT for various cancers**

NIR-PIT can be applied with any surface marker of cancer or stromal cells provided that an antibody exists to bind to it [8, 58, 59]. In this section, we discuss a range of NIR-PIT applications for various cancers in clinical and pre-clinical studies (Figure 3).

**4.1 Head and neck squamous cell carcinoma**

Head and neck cancer is the 7th most common cancer with >931,000 new cases worldwide and is 7th the most common cause of cancer death with >467,000 deaths worldwide in 2020 [2]. Head and neck squamous cell carcinoma (HNSCCs) is the most common type of head and neck cancer [60]. Risk factors for HNSCCs are exposure to tobacco-derived carcinogens, excessive alcohol consumption, and human papillomavirus or Epstein–Barr virus infection. Approximately 30-40% of HNSCC patients present at an early stage and are curable with surgery or radiotherapy alone. However, multimodality treatment including surgery, radiation, chemotherapy, or immunotherapy is often required for late-stage patients, which constitute more than 60% of patients [61]. The side effects of these combined therapies can damage the delicate structures controlling speech, taste and swallowing leading to debilitating loss of quality of life [62]. Therefore, developing new therapeutic methods which treat the cancer effectively while preserving function is a high priority.

Head and neck cancers are amenable to NIR-PIT because they are often close to the skin or mucosa. Interstitial light fibers can be inserted into deeper tumors. Because head and neck cancers can invade critical vascular structures, care must be taken when treating head and neck cancers with NIR-PIT.

The most clinical experience exists for head and neck NIR-PIT. Epithelial Growth Factor Receptor (EGFR) is overexpressed in up to 90% of HNSCCs [63]. Cetuximab, which is a chimeric IgG1 monoclonal antibody and a competitive inhibitor of EGFR ligand binding, was approved by the FDA in 2006. Cetuximab-IR700 was the first agent clinically introduced for NIR-PIT. A Phase 1/2 clinical trial of NIR-PIT using cetuximab-IR700 in patients with recurrent HNSCC concluded in 2017 and showed that cetuximab-IR700 NIR-PIT is more effective than current second- and third-line therapies for recurrent HNSCCs [64]. These results prompted the FDA to assign a fast track designation for cetuximab-IR700 in 2018. In September 2020, cetuximab-IR700 received conditional approval from the Japanese Ministry of Health, Labor and Welfare as a treatment for HNSCC patients. A global phase 3 trial in recurrent HNSCC is currently underway [35]. It is anticipated that NIR-PIT may be used earlier in the disease, including on premalignant lesions in the mouth such as leukoplakia which also express EGFR.

CD44, a cancer stem cell marker, is also expressed on HNSCC. Its presence is a negative prognostic indicator and is associated with tumor progression, metastasis and poor prognosis [65]. In mouse homograft models, CD44 targeted NIR-PIT suppressed tumor growth and prolonged survival [32]. Moreover, CD44 targeted NIR-PIT with PD-1 blockade therapy was more effective than single therapies in mouse homograft models including a minimally immunogenic tumor [31, 34].

**4.2 Glioblastoma multiforme**

Glioblastoma multiforme (GBM) is among the most aggressive tumors in adults and carries a dismal 5 year prognosis of only 5.5% [66, 67]. In the United States, an estimated 11,833 patients are diagnosed with GBM per year [68]. The current standard of care for patients younger than age 70 years with newly diagnosed GBM is maximal safe surgical resection, followed by radiation therapy and concomitant temozolomide (TMZ) followed by adjuvant TMZ [66]. For elderly patients performance status can affect treatment decisions and is adjusted according to the ability of the patient to tolerate the intense combination therapy [69]. Even though GBM is well known to extend beyond the visible borders seen on brain MRI, studies of recurrence patterns of GBM after surgery, radiotherapy, and chemotherapy have shown that ∼80–90% of recurrences are within the original treatment field [70-73], suggesting the efficacy of current therapeutic methods is insufficient. NIR-PIT could be a useful adjuvant to surgery to selectively kill the unresected tumor cells that invade around surgical cavity.

Although NIR light can transmit through the skull it is uncertain whether it is of sufficient intensity to treat GBMs. More likely NIR-PIT will be used with thin fibro optic diffusers through inserted small catheter or as an adjuvant to surgery after the skull is opened. Direct light application to the surgical field could be useful in sterilizing tumor margins. It is possible that wireless LEDs could also be inserted in the surgical cavity to supply light as needed in case of recurrent disease [22].

Several studies have shown EGFR gene amplification in ∼40% of all GBMs [74-76]. Burley et al. reported that EGFR targeted NIR-PIT showed effectiveness in xenograft models of GBM [77]. Therefore, the most likely first APC to be used in GBM will be cetuximab-IR700.

Therapeutic resistance may also arise from cancer stem cells (CSC) within GBMs [78, 79]. The neural stem cell marker CD133, has been the CSC marker most associated with GBM, and identifies cells with higher rates of self-renewal and proliferation and increased differentiation ability [80, 81]. In orthotopic xenograft models, anti-CD133-IR700 conjugates accumulated in brain tumors, suggesting anti-CD133-IR700 can pass through the blood brain barrier. Moreover, CD133 targeted NIR-PIT was highly efficient in both the subcutaneous and orthotopic models [11]. Therefore, EGFR or CSC-targeted NIR-PIT is a potential therapy for GBMs.

**4.3** **Esophageal cancer**

Esophageal carcinoma is the 11th most common cancer with >604,000 cases worldwide and is the 6th most common cause of cancer death with >544,000 deaths worldwide in 2020 [2]. The two major subtypes of esophageal cancer are esophageal squamous cell carcinoma (ESCC) and esophageal adenocarcinoma (EAC). ESCC represents 90% of all cases of esophageal cancer globally and is dominant in East Asia, East Africa, and South America. EAC is more common in developed countries than in developing countries [82]. Most patients with esophageal cancer need multimodality treatment, including chemotherapy, chemoradiotherapy and/or surgical resection. Recurrences are particularly difficult to treat.

NIR-PIT would most likely be applied in conjunction with upper gastrointestinal (GI) endoscopy. The operator could identify the tumor location and then apply NIR light to tumors in patients who had previously received the APC. Naturally, treatment of transmural tumors could lead to esophageal perforations so proper patient selection is needed.

Between 71-91% of ESCCs and 32-64% of EACs express EGFR and therefore may be potential candidates for EGFR-targeted NIR-PIT [83-87]. Overexpression of HER2 has been reported in up to 64% of ESCCs and 32% of EACs, respectively [88-90]. In vitro studies, EGFR or HER2 targeted NIR-PIT has been shown to be effective in esophageal carcinoma cell lines [91].

In addition to epithelial growth factor receptors, many studies have emphasized the importance of cancer-associated fibroblasts (CAFs) in esophageal carcinoma [92, 93]. CAF targeted NIR-PIT inhibited tumor progression in co-culture models of ESCCs and CAFs [94, 95]. Therefore, esophageal cancer could be treated with some combination of EGFR-, HER2- or CAF- targeted NIR-PIT. It is possible in the future that cocktails of APCs could be used to more completely treat particular cancers.

**4.4 Lung cancer**

Lung cancer is the 2nd most common cancer with >2,206,000 new cases worldwide and is the most common cause of cancer death with >1,796,000 deaths worldwide in 2020 [2]. The two major subtypes of lung cancer are non-small cell lung cancer NSCLC (85% of patients) and small cell lung cancer (SCLC) (15%) [96].

The lungs are a particularly interesting organ to consider treating with NIR-PIT. NIR light transmits through air very well. For lesions near the main bronchi it may be possible to deliver light via a bronchoscope. For deeper lesions a bronchoscope or transcutaneous fiber might be used. It may be possible to treat several lesions simultaneously by applying light to normal lung and having it transmit throughout the lung. Intraoperatively, light could be directed to pleural surfaces or to the lung parenchyma.

EGFR overexpression has been identified in 40-80% of NCSLCs [97]. NIR-PIT with panitumumab (another antibody targeting EGFR)-IR700 conjugates inhibited tumor growth in a transgenic mouse model of spontaneous lung cancer expressing human EGFR [15]. Several mAbs against programmed cell death protein-1 ligand (PD-L1) and PD-1 have demonstrated clinical benefit in patients with NSCLC and are collectively known as checkpoint inhibitors [98-100]. PD-L1 is over expressed in many cancers, and therefore it is a potential target for NIR-PIT. NIR-PIT using avelumab (human anti-PD-L1 mAb)-IR700 induced significant therapeutic effects in an NSCLC xenograft model [101]. Thus, PD-L1 targeted NIR-PIT might be useful for NSCLC cancers with high PD-L1 expression. SCLC has a poor prognosis and it is commonly diagnosed at an advanced, unresectable stage [102]. Delta-like protein 3 (DLL3) is a potential therapeutic target molecule for SCLC [103], but rovalpituzumab tesirine, which is the first antibody drug conjugate (ADC) targeting DLL3, was terminated on August 2019 because of failure of both the TAHOE (NCT03061812) and MERU (NCT03033511) clinical trials. The failure of this ADC is not necessarily an indictment of the antibody, however. NIR-PIT targeting DDL3 showed marked antitumor effects [104]. Malignant cells in the pleural fluid or pleural metastases are classified as M1a and stage IV according to TNM Classification of Malignant Tumors 8th edition (UICC 8th edition) and typically surgical resection is excluded [105]. Therefore, therapies that could treat pleural metastases without damage to the adjacent organs might prolong survival. In xenograft models, HER2 targeted NIR-PIT led to significant reduction in pleural dissemination by HER2 expressing NSCLC cells [18]. Furthermore, in mouse models of lung metastasis, HER2 targeted NIR-PIT showed significant reductions in metastasis tumor volume and prolonged survival [16, 17]. These results suggest a potential new therapy for the local control of lung metastases or pleural dissemination which could readily be translated to clinical treatments.

**4.5 Malignant pleural mesothelioma**

Malignant pleural mesothelioma (MPM) is a malignant tumor that originates from mesothelial cells in the pleura and peritoneum and has an extremely poor prognosis, with a median survival of 8–14 months [106]. It is often debulked with extrapleural pneumonectomies but recurrence is common.

NIR-PIT could be considered at the time of the debulking procedure. At that time the mesothelioma would be exposed and NIR light could be directly applied to the tumor surface. It may also be possible to deliver light to the pleura broncho-scopically in some cases.

Podoplanin (PDPN) is a type I transmembrane glycoprotein that is expressed in lymphatic endothelial cells, type I alveolar epithelial cells, and podocytes of the glomeruli. PDPN is a specific pathological diagnostic marker to distinguish lymphatic vessels from blood vessels but it is also expressed in MPM [107-109]. PDPN targeted NIR-PIT in MPM models suppressed tumor progression [110]. Another MSM marker, mesothelin (MSLN) is a cell surface glycoprotein that is a target for antibody-based therapies [111, 112]. MSLN targeted NIR-PIT has been shown to be effective in mouse xenograft models [113]. These findings suggest that PDPN and/or MSLN targeted NIR-PIT might be a potential alternative treatment of MPM.

**4.6 Breast cancer**

Female breast cancer is the most common cancer with >2,260,000 new cases worldwide and is the 5th most common cause of cancer death with >684,000 deaths worldwide in 2020 [2]. Breast cancer is classified into 3 major subtypes based on the presence or absence of molecular markers for two hormone receptors (HR) and human epidermal growth factor 2 (HER2). HER2, which is a member of the epidermal growth factor receptor family, regulates cell proliferation, differentiation, and apoptosis through signal transduction. Tumors are classified by their HR and HER2 status: HR+/HER2− (70% of patients), HR±/HER2+ (15%-20%), and “triple-negative” (HR−/HER2−; 10-15%) [114]. Breast cancer is treated by multimodal therapy. Locoregional therapies include surgery and radiation therapy. Systemic therapies include hormone therapy for HR+ patients, chemotherapy , HER2-targeted therapy for HER2+ patients, bone-modifying agents, Poly(ADP-ribose) polymerase (PARP) inhibitors for BRCA-mutated cancer and immunotherapy [114-117].

NIR-PIT could be used in a variety of ways in breast cancer. For localized disease it could be a method of treating the cancer with interstitial fibers. For local recurrences or recurrences in the chest wall a similar strategy could be used. For lung metastases it might be possible to illuminate the lungs to deliver light.

Trastuzumab, an anti-HER2 mAb has been used as an NIR-PIT agent in xenograft models of breast cancer [7]. Cells that are unresponsive to HER2 targeted NIR-PIT are often shown to be HER2 targeted NIR-PIT-responsive after viral transduction of the HER2-extracellular domain [118]. For triple-negative breast tumors, no targeted therapy is currently available. However, EGFR expression has been reported in 50-89% of cases [119, 120]. Therefore, EGFR targeted NIR PIT could be used in some cases of triple-negative breast cancer. In xenograft models with two different cell lines established from triple-negative breast cancers, cetuximab-IR700 NIR-PIT suppressed tumor growth and prolonged survival [121].

**4.7 Gastric cancer**

Gastric cancer is the 6th most common cancer with >1,089,000 new cases worldwide and is the 4th most common cause of cancer death with >768,000 deaths worldwide in 2020 [2]. *H. pylori* infection has been implicated in more than 93% of gastric cancer patients [122]. In the Trastuzumab for Gastric Cancer trial (ToGA trial), HER2 positivity was 22.1% [89]. The FDA approved Trastuzumab (anti-HER2 mAb) in combination with chemotherapy as a standard treatment for patients with HER2+ advanced gastric or gastro-esophageal junction cancer in 2010 [123]. In mouse models of peritoneal carcinomatosis or a flank tumor, HER2 targeted NIR-PIT showed significant reductions in tumor volume [124]. The combination therapy of HER2 targeted NIR-PIT (using Trastuzumab-IR700) and conventional chemotherapy of 5-FU rapidly induced significant tumor inhibition [125]. Currently, anti-HER2 antibodies recognizing different epitopes of HER2 have been developed, such as pertuzumab which could also be used for NIR-PIT. NIR-PIT with trastuzumab-IR700 and pertuzumab-IR700 conjugates showed stronger antitumor effects than either antibody conjugate alone [126]. Less than 6% of the normal gastric tissues demonstrated EGFR expression, whereas EGFR was expressed in 41.8–57.7% of gastric cancers by immunohistochemistry (IHC) analysis [127-129]. Therefore, EGFR targeted NIR-PIT, perhaps in combination with HER2-targeted NIR-PIT might be a suitable treatment for some patients with gastric cancer.

**4.8 Colorectal Cancer**

Colorectal cancer is the 3rd most common cancer with >1,880,000 new cases worldwide and is the 2nd most common cause of cancer death with >915,000 deaths worldwide in 2020. The incidence rate of colorectal cancer is higher in developed countries than in developing countries [2]. With economic growth in developing countries, it is estimated that the incidence of colorectal cancer could increase to 2.5 million new cases by 2035 [130]. Carcinoembryonic antigen (CEA) is preferentially expressed in colon cancer cells compared to normal colon cells [131, 132]. Moreover, overexpression of CEA in tumor tissue is associated is a negative prognostic sign [133, 134]. CEA targeted NIR-PIT inhibited tumor growth in a CEA-expressing mouse xenograft model [135, 136].

Another potential target for NIR-PIT in colon cancer is the Glycoprotein A33 antigen (GPA33) which is highly expressed in over 95% of human colorectal cancers and exhibits limited expression in normal intestinal epithelium [137]. GPA33 targeted NIR-PIT showed significant efficacy in xenograft models [138]. EGFR overexpression has been observed in 43.9–97% of colorectal cancer patients based on IHC analysis [139-142]. Some of the anti-EGFR mAbs including cetuximab and panitumumab have been approved by FDA as first-line treatments of colorectal cancer [143]. Thus, EGFR targeted NIR-PIT could be utilized as adjuvant therapy in conjunction with surgery or laparoscopy.

**4.9 Liver cancer**

Liver cancer is the 8th most common with >905,000 new cases worldwide and is the 3rd most common cause of cancer death with >830,000 deaths worldwide in 2020 [2]. Hepatocellular carcinoma (HCC) is the most common type of primary hepatic malignancy. HCC is caused by chronic viral hepatitis which results in cirrhosis and tumor formation. Treatments include surgery, radiation, chemotherapy, immunotherapy, and liver transplantation. In general, mortality rates are high for HCC.

Since HCC is usually found within the liver parenchyma it would have to be approached with catheters or fiber optic needles. The liver itself is relatively poor at light transmission so it is important that the fiber optic needles would be placed within the tumor itself. In the future it may be possible to leave in place wireless LEDs to provide light in the event of recurrence after NIR-PIT.

Glypican-3 (GPC3) is highly expressed in HCC but not in normal tissue, and therefore, is a target-candidate for NIR-PIT [144]. GPC3 targeted NIR-PIT inhibited tumor growth compared to untreated controls in a xenograft model of HCC [145]. Moreover, the combination of GPC3 targeted NIR-PIT and nanoparticle albumin-bound paclitaxel enhanced the therapeutic effect compared to either alone [57]. The post NIR-PIT SUPR effect enabled more drug delivery to the tumor. Cholangiocarcinoma (CCA) is the second most common liver cancer after HCC accounting for ∼15% of all primary liver cancers and ∼3% of all gastrointestinal cancers worldwide [146]. It is difficult to deliver NIR light into the bile duct for CCA from outside the body. However, newly developed catheter-based devices containing LEDs could be used to deliver light for NIR-PIT resulting in tumor suppression in xenograft models [147]. Tumor-associated calcium signal transducer 2 (TROP2) is overexpressed in many epithelial cancers including CCA [148] and TROP2 corelates with a poor prognosis in various cancers [149]. TROP2 targeted NIR-PIT inhibited tumor growth in CCA xenograft model [150].

**4.10 Pancreatic cancer**

Pancreatic cancer (PC) is the 14th most common cancer with >495,000 new cases worldwide and the 8th most common cause of cancer death with >466,000 deaths worldwide in 2020 [2]. Pancreatic cancer is aggressive and often presents late because symptoms are nonspecific or minimal early in the disease. The pancreatic cancer cell is particularly aggressive and the tumor recurrence rate after radical surgical resection is 80% even though surgical techniques and adjuvant treatments have improved in the last decades [151].

The pancreas primary is difficult to approach with light. It is possible, depending on the tumor location in the pancreas, that endoscopic-ultrasound-light delivery could provide light to the tumor. More realistically, NIR-PIT could be performed as an adjuvant to open or laparoscopic surgery. Given the high rate of recurrence it may be especially beneficial to have a wide field of NIR-PIT in pancreatic cancer.

Among the targets potentially suitable for NIR-PIT targeting is anti-CEA mAb-IR700 which demonstrated a good response in an orthotopic xenograft model [152]. Cadherin-17 (CDH17) is highly expressed on gastrointestinal cancer cells. CDH17 targeted NIR-PIT inhibited tumor growth in a xenograft model which used a pancreatic cancer cell line [153]. TROP2 is also overexpressed in PC [148]. TROP2 targeted NIR-PIT inhibited tumor growth in PC xenograft models [150]. Moreover, CEA-targeted NIR-PIT following surgery reduced recurrence by eliminating remaining cancer cells [154, 155]. EGFR expression was observed in 62%-69% of PC patients with IHC analysis [156-158]. Thus, some combination of antibody conjugates engaging CEA, CDH17, TROP2 and EGFR might be successful in pancreatic cancer.

**4.11 Ovarian cancer**

Ovarian cancer is the 19th most common cancer with >313,000 new cases worldwide and the 15th most common cause of cancer death with >207,000 deaths worldwide in 2020 [2]. Since ovarian cancer is a highly metastatic disease, a minority (15%) of patients are diagnosed with localized tumor (stage I) however, when early diagnosis can be made the 5-year survival is 92%. However, the majority of cases present late with disseminated intra-abdominal disease (stages III–IV) with a 5-year survival of 25% [159, 160]. Standard therapy of ovarian cancer is a combination of chemotherapy and surgery. Cytoreductive surgery is initially employed to remove all macroscopic disease (R0 resection). The success of the R0 resection is a prognostic indicator [161, 162]. When the disease is considered to be R1 (< 1 cm), all visible disease is removed but viable microscopic cancer cells are assumed to remain at the surgical margin and recurrence is common [160, 163]. Therefore, the development of new therapies for treating residual disease in the peritoneum after cytoreductive surgery is needed.

A typical pattern of recurrence is in the peritoneum where direct light exposure is possible during open or laparoscopic procedures. Lymph node metastases present a greater problem and may require direct light exposure during surgery. While NIR-PIT might not be curative in all cases it could be very useful in killing recurrent peritoneal disease thus staving off the primary symptom of recurrent ovarian cancer, malignant ascites.

HER2 targeted NIR-PIT showed significant tumor suppression in subcutaneous tumor models but also in disseminated peritoneal models using HER2 expressing ovarian cancer cell lines [164]. Galactosyl serum albumin (GSA) binds to beta-D-galactose receptors, which is overexpressed on the surface of many ovarian tumors [165]. GSA targeted NIR-PIT specifically killed ovarian cancer cells (SHIN3) in vitro and suppressed tumor growth in a peritoneal disseminated model [166].

**4.12 Bladder cancer**

Bladder cancer is the 13th most common cancer with >573,000 new cases and is the 14th most common cause of cancer death with >212,000 deaths worldwide in 2020 [2]. Evaluation of bladder cancer patients is performed using cystoscopy with a flexible scope [167]. EGFR and HER2 were detected in 72.2% and 44.5% of bladder cancers, respectively [168]. Therefore, these receptors could be targets for NIR-PIT in bladder cancer. EGFR targeted NIR-PIT caused cell death in human bladder tumor cell lines in vitro and inhibited tumor growth in bladder tumor xenograft models [169, 170]. Moreover, combined EGFR and HER2 targeted NIR-PIT inhibited tumor growth significantly in a xenograft bladder tumor model [171]. CD47, one of the “don't eat me” signals for macrophages, is also highly expressed (80%) in bladder cancer tumors, but is not expressed on normal luminal urothelium [172]. Kiss et al. reported that CD47 targeted NIR-PIT killed human bladder tumor cell lines and patient derived bladder tumor cells in vivo, and suppressed tumor growth in xenograft models [173].

**4.13 Prostate cancer**

Prostate cancer is the 4th most common cancer with >1,414,000 new cases and is the 9th most common cause of cancer death with >375,000 deaths worldwide in 2020 [2]. Prostate cancer is treated by surgery, radiation or active surveillance in the case of low-grade cancers. Because of the high morbidity of prostate cancer treatments (urinary incontinence and erectile dysfunction) alternative therapies have been proposed. Focal therapies consist of ablative methods that physically destroy prostate cancers but also tend to undertreat infiltrative disease while damaging normal prostate tissue.

Prostate cancer NIR-PIT could be relatively straightforward as the prostate is commonly biopsied using MRI-Ultrasound fusion imaging. Instead of biopsy needles, fiber optic needles could be introduced into the prostate enabling the killing of cancer cells while sparing critical structures like the urethra, sphincters and pelvic nerves.

Prostate-specific membrane antigen (PSMA) is overexpressed significantly in prostatic cancer cells and the expression level of PSMA is associated with the stage and grade of the prostate cancer but the expression is low in normal tissues [174]. Thus, PSMA is a reasonable target for molecular therapy. PSMA targeted NIR-PIT eliminated prostate tumor cells significantly in vivo and suppressed tumor progression and prolonged survival in xenograft models [175]. Moreover, PSMA targeted NIR-PIT using anti-PSMA diabody (Db) or anti-PSMA minibody (Mb), which are small and bivalent antibody fragments of anti-PSMA-IgG, showed PSMA+ cell death in vitro and suppressed tumor growth in a xenograft model [176]. A major advantage of PSMA-targeted NIR-PIT is that suitable patients could be determined using PSMA PET scans prior to the procedure [177]. This would localize the tumor accurately and allow placement of fiber optic catheters. Following NIR-PIT the same PSMA PET scan could be used to determine the success of the procedure and whether additional treatments might be needed.

**4.14 Lymphoma**

Lymphoma is the 9th most common cancer with >627,000 new cases worldwide and is the 12th most common cause of cancer death with >283,000 deaths worldwide in 2020 [2]. Traditionally, Lymphoma is divided into Hodgkin’s lymphoma (approx. 13% of all lymphomas) and non-Hodgkin lymphoma [2]. The majority of Lymphomas are B cell origin [178]. B-cell lymphomas often express B-cell markers, such as CD19 and CD20, which can bind specific monoclonal antibodies [179]. CD20 targeted NIR-PIT using rituximab-IR700 conjugates showed efficacy in xenograft models of B-cell lymphoma[180]. Furthermore, the therapeutic effect of CD20 targeted NIR-PIT was more effective than that of radioimmunotherapy in a xenograft model of aggressive B-cell lymphoma [181]. Mycosis fungoides (MF) is a rare cancer, however it is the most common subtype of cutaneous T-cell lymphoma [182]. It is reported that MF cells express cutaneous lymphocyte antigen (CLA) [183]. CLA targeted NIR-PIT specifically killed MF cell line in vitro [184]. Thus, NIR-PIT could treat the locoregional lymphomas including skin-based lymphoma which would be highly amenable to direct NIR light exposure.

**4.15 Melanoma**

Melanoma is the most aggressive and the lethal form of skin cancer. Melanoma is treated by multimodal therapies, such as surgical resection, chemotherapy, photodynamic therapy, immunotherapy, and targeted therapy using small molecule inhibitors or antibodies [185].

Because early melanoma is usually present on the skin surface it is relatively amenable to direct NIR light exposure, however, deeper nodal involvement might require interstitial placement of catheters/fiber optics in order to delivery light to all facets of the tumor.

CD146 has been identified as a melanoma cell adhesion molecule. CD146 is overexpressed in 70% of primary melanomas and 90% of lymph node metastases [186]. Wei et al. reported that CD146 targeted NIR-PIT inhibited tumor growth in CD146-positive melanoma xenograft model [187]. Thus, antiCD146-targeted NIR-PIT could be a potential method of treating melanomas without highly disfiguring surgery.

**4.16 Bone metastases**

Bone is the 3rd most common site of metastases in cancer patients [188]. It has been assumed that NIR-PIT for bone metastases would have no effect because light cannot penetrate bone. However, ex vivo experiments showed that NIR-PIT not only penetrates bone but can kill tumor cells located behind bone. Moreover, tumor viability was reduced by NIR-PIT [189]. Hence, in spite of decreasing of light transmittance, NIR-PIT nonetheless is able to treat cancers within bone.

**5. Conclusion**

NIR-PIT is a new cancer therapy with broad applications. It has an immediate effect on the tumor neovasculature which results in the SUPR effect which enables nanodrugs to penetrate into the treated tumor at far higher concentrations than are normally possible. It also profoundly activates the immune system both locally and, in some cases, systemically. NIR-PIT kills cancers in a highly specific manner and therefore, could be used in a variety of cancers. Each cancer requires one or more specific antibodies that bind the tumor and can be conjugated with IR700 to become an NIR-PIT agent. Using cocktails of mAb-IR700 conjugates that are injected intravenously and various methods of delivering light, a wide range of tumors could be treated with minimal side effects. Recently, EGFR targeted NIR-PIT was conditionally approved in Japan and a phase 3 clinical trial is ongoing. Thus, NIR-PIT has great potential to become a widely applicable cancer therapy in the near future.

**References**

[1] Cancer. WHO, 2018. (Accessed February 26, 2021, at <https://www.who.int/news-room/fact-sheets/detail/cancer>.)

[2] Sung H, Ferlay J, Siegel RL, et al. Global cancer statistics 2020: GLOBOCAN estimates of incidence and mortality worldwide for 36 cancers in 185 countries. CA Cancer J Clin 2021

[3] Robert C. A decade of immune-checkpoint inhibitors in cancer therapy. Nat Commun 2020,11,3801

[4] Galon J, Bruni D. Approaches to treat immune hot, altered and cold tumours with combination immunotherapies. Nat Rev Drug Discov 2019,18,197-218

[5] Bonaventura P, Shekarian T, Alcazer V, et al. Cold Tumors: A Therapeutic Challenge for Immunotherapy. Front Immunol 2019,10,168

[6] Dubbs SB. The Latest Cancer Agents and Their Complications. Emerg Med Clin North Am 2018,36,485-92

[7] Mitsunaga M, Ogawa M, Kosaka N, Rosenblum LT, Choyke PL, Kobayashi H. Cancer cell-selective in vivo near infrared photoimmunotherapy targeting specific membrane molecules. Nat Med 2011,17,1685-91

[8] Kobayashi H, Choyke PL. Near-Infrared Photoimmunotherapy of Cancer. Acc Chem Res 2019,52,2332-9

[9] Sato K, Nakajima T, Choyke PL, Kobayashi H. Selective cell elimination in vitro and in vivo from tissues and tumors using antibodies conjugated with a near infrared phthalocyanine. RSC Adv 2015,5,25105-14

[10] Sato K, Sato N, Xu B, et al. Spatially selective depletion of tumor-associated regulatory T cells with near-infrared photoimmunotherapy. Sci Transl Med 2016,8,352ra110

[11] Jing H, Weidensteiner C, Reichardt W, et al. Imaging and Selective Elimination of Glioblastoma Stem Cells with Theranostic Near-Infrared-Labeled CD133-Specific Antibodies. Theranostics 2016,6,862-74

[12] Sato K, Ando K, Okuyama S, et al. Photoinduced Ligand Release from a Silicon Phthalocyanine Dye Conjugated with Monoclonal Antibodies: A Mechanism of Cancer Cell Cytotoxicity after Near-Infrared Photoimmunotherapy. ACS Cent Sci 2018,4,1559-69

[13] Ogata F, Nagaya T, Okuyama S, et al. Dynamic changes in the cell membrane on three dimensional low coherent quantitative phase microscopy (3D LC-QPM) after treatment with the near infrared photoimmunotherapy. Oncotarget 2017,8,104295-302

[14] Henderson TA, Morries LD. Near-infrared photonic energy penetration: can infrared phototherapy effectively reach the human brain? Neuropsychiatr Dis Treat 2015,11,2191-208

[15] Nakamura Y, Ohler ZW, Householder D, et al. Near Infrared Photoimmunotherapy in a Transgenic Mouse Model of Spontaneous Epidermal Growth Factor Receptor (EGFR)-expressing Lung Cancer. Mol Cancer Ther 2017,16,408-14

[16] Sato K, Nagaya T, Nakamura Y, Harada T, Choyke PL, Kobayashi H. Near infrared photoimmunotherapy prevents lung cancer metastases in a murine model. Oncotarget 2015,6,19747-58

[17] Sato K, Nagaya T, Mitsunaga M, Choyke PL, Kobayashi H. Near infrared photoimmunotherapy for lung metastases. Cancer Lett 2015,365,112-21

[18] Sato K, Nagaya T, Choyke PL, Kobayashi H. Near infrared photoimmunotherapy in the treatment of pleural disseminated NSCLC: preclinical experience. Theranostics 2015,5,698-709

[19] Maruoka Y, Nagaya T, Sato K, et al. Near Infrared Photoimmunotherapy with Combined Exposure of External and Interstitial Light Sources. Mol Pharm 2018,15,3634-41

[20] Nagaya T, Okuyama S, Ogata F, Maruoka Y, Choyke PL, Kobayashi H. Endoscopic near infrared photoimmunotherapy using a fiber optic diffuser for peritoneal dissemination of gastric cancer. Cancer Sci 2018,109,1902-8

[21] Okuyama S, Nagaya T, Sato K, et al. Interstitial near-infrared photoimmunotherapy: effective treatment areas and light doses needed for use with fiber optic diffusers. Oncotarget 2018,9,11159

[22] Nakajima K, Kimura T, Takakura H, et al. Implantable wireless powered light emitting diode (LED) for near-infrared photoimmunotherapy: device development and experimental assessment in vitro and in vivo. Oncotarget 2018,9,20048-57

[23] Inagaki FF, Fujimura D, Furusawa A, et al. Diagnostic imaging in near-infrared photoimmunotherapy using a commercially available camera for indocyanine green. Cancer Sci 2021

[24] Ogawa M, Tomita Y, Nakamura Y, et al. Immunogenic cancer cell death selectively induced by near infrared photoimmunotherapy initiates host tumor immunity. Oncotarget 2017,8,10425-36

[25] Kobayashi H, Furusawa A, Rosenberg A, Choyke PL. Near-infrared photoimmunotherapy of cancer: a new approach that kills cancer cells and enhances anti-cancer host immunity. Int Immunol 2021,33,7-15

[26] Green DR, Ferguson T, Zitvogel L, Kroemer G. Immunogenic and tolerogenic cell death. Nat Rev Immunol 2009,9,353-63

[27] Kroemer G, Galluzzi L, Kepp O, Zitvogel L. Immunogenic cell death in cancer therapy. Annu Rev Immunol 2013,31,51-72

[28] Galluzzi L, Vitale I, Abrams JM, et al. Molecular definitions of cell death subroutines: recommendations of the Nomenclature Committee on Cell Death 2012. Cell Death Differ 2012,19,107-20

[29] Krysko DV, Garg AD, Kaczmarek A, Krysko O, Agostinis P, Vandenabeele P. Immunogenic cell death and DAMPs in cancer therapy. Nat Rev Cancer 2012,12,860-75

[30] Galluzzi L, Buque A, Kepp O, Zitvogel L, Kroemer G. Immunogenic cell death in cancer and infectious disease. Nat Rev Immunol 2017,17,97-111

[31] Nagaya T, Friedman J, Maruoka Y, et al. Host Immunity Following Near-Infrared Photoimmunotherapy Is Enhanced with PD-1 Checkpoint Blockade to Eradicate Established Antigenic Tumors. Cancer Immunol Res 2019,7,401-13

[32] Nagaya T, Nakamura Y, Okuyama S, et al. Syngeneic Mouse Models of Oral Cancer Are Effectively Targeted by Anti-CD44-Based NIR-PIT. Mol Cancer Res 2017,15,1667-77

[33] Maruoka Y, Furusawa A, Okada R, et al. Near-Infrared Photoimmunotherapy Combined with CTLA4 Checkpoint Blockade in Syngeneic Mouse Cancer Models. Vaccines (Basel) 2020,8

[34] Wakiyama H, Furusawa A, Okada R, et al. Increased Immunogenicity of a Minimally Immunogenic Tumor after Cancer-Targeting Near Infrared Photoimmunotherapy. Cancers (Basel) 2020,12

[35] ASP-1929 Photoimmunotherapy (PIT) Study in Recurrent Head/Neck Cancer for Patients Who Have Failed at Least Two Lines of Therapy. ClinicalTrials.gov, 2020. (Accessed February 26, 2021, at <https://clinicaltrials.gov/ct2/show/NCT03769506>.)

[36] Oun R, Moussa YE, Wheate NJ. The side effects of platinum-based chemotherapy drugs: a review for chemists. Dalton Trans 2018,47,6645-53

[37] Ramirez LY, Huestis SE, Yap TY, Zyzanski S, Drotar D, Kodish E. Potential chemotherapy side effects: what do oncologists tell parents? Pediatr Blood Cancer 2009,52,497-502

[38] Golombek SK, May JN, Theek B, et al. Tumor targeting via EPR: Strategies to enhance patient responses. Adv Drug Deliv Rev 2018,130,17-38

[39] Matsumura Y, Maeda H. A new concept for macromolecular therapeutics in cancer chemotherapy: mechanism of tumoritropic accumulation of proteins and the antitumor agent smancs. Cancer Res 1986,46,6387-92

[40] Maeda H, Wu J, Sawa T, Matsumura Y, Hori K. Tumor vascular permeability and the EPR effect in macromolecular therapeutics: a review. Journal of Controlled Release 2000,65,271-84

[41] Petersen GH, Alzghari SK, Chee W, Sankari SS, La-Beck NM. Meta-analysis of clinical and preclinical studies comparing the anticancer efficacy of liposomal versus conventional non-liposomal doxorubicin. Journal of Controlled Release 2016,232,255-64

[42] Anselmo AC, Mitragotri S. Nanoparticles in the clinic: An update. Bioeng Transl Med 2019,4,e10143

[43] Ventola CL. Progress in Nanomedicine: Approved and Investigational Nanodrugs. P T 2017,42,742-55

[44] Nakamura Y, Mochida A, Choyke PL, Kobayashi H. Nanodrug Delivery: Is the Enhanced Permeability and Retention Effect Sufficient for Curing Cancer? Bioconjug Chem 2016,27,2225-38

[45] Manoukian G, Hagemeister F. Denileukin diftitox: a novel immunotoxin. Expert Opin Biol Ther 2009,9,1445-51

[46] Green DJ, Press OW. Whither Radioimmunotherapy: To Be or Not To Be? Cancer Res 2017,77,2191-6

[47] Wang Y, Kohane DS. External triggering and triggered targeting strategies for drug delivery. Nature Reviews Materials 2017,2,17020

[48] Ta T, Porter TM. Thermosensitive liposomes for localized delivery and triggered release of chemotherapy. J Control Release 2013,169,112-25

[49] Bae Y, Nishiyama N, Fukushima S, Koyama H, Yasuhiro M, Kataoka K. Preparation and Biological Characterization of Polymeric Micelle Drug Carriers with Intracellular pH-Triggered Drug Release Property:  Tumor Permeability, Controlled Subcellular Drug Distribution, and Enhanced in Vivo Antitumor Efficacy. Bioconjugate Chemistry 2005,16,122-30

[50] Jain RK. Molecular regulation of vessel maturation. Nature Medicine 2003,9,685-93

[51] Dewhirst MW, Secomb TW. Transport of drugs from blood vessels to tumour tissue. Nat Rev Cancer 2017,17,738-50

[52] Stylianopoulos T, Martin JD, Chauhan VP, et al. Causes, consequences, and remedies for growth-induced solid stress in murine and human tumors. Proc Natl Acad Sci U S A 2012,109,15101-8

[53] Inagaki FF, Furusawa A, Choyke PL, Kobayashi H. Enhanced nanodrug delivery in tumors after near-infrared photoimmunotherapy. Nanophotonics 2019,8,1673-88

[54] Sano K, Nakajima T, Choyke PL, Kobayashi H. Markedly enhanced permeability and retention effects induced by photo-immunotherapy of tumors. ACS nano 2013,7,717-24

[55] Kobayashi H, Choyke PL. Super enhanced permeability and retention (SUPR) effects in tumors following near infrared photoimmunotherapy. Nanoscale 2016,8,12504-9

[56] Sano K, Nakajima T, Choyke PL, Kobayashi H. The effect of photoimmunotherapy followed by liposomal daunorubicin in a mixed tumor model: a demonstration of the super-enhanced permeability and retention effect after photoimmunotherapy. Mol Cancer Ther 2014,13,426-32

[57] Hanaoka H, Nakajima T, Sato K, et al. Photoimmunotherapy of hepatocellular carcinoma-targeting Glypican-3 combined with nanosized albumin-bound paclitaxel. Nanomedicine (Lond) 2015,10,1139-47

[58] Okada R, Maruoka Y, Furusawa A, et al. The Effect of Antibody Fragments on CD25 Targeted Regulatory T Cell Near-Infrared Photoimmunotherapy. Bioconjug Chem 2019,30,2624-33

[59] Okada R, Kato T, Furusawa A, et al. Local Depletion of Immune Checkpoint Ligand CTLA4 Expressing Cells in Tumor Beds Enhances Antitumor Host Immunity. Advanced Therapeutics 2021,n/a,2000269

[60] Johnson DE, Burtness B, Leemans CR, Lui VWY, Bauman JE, Grandis JR. Head and neck squamous cell carcinoma. Nat Rev Dis Primers 2020,6,92

[61] Chow LQM. Head and Neck Cancer. N Engl J Med 2020,382,60-72

[62] List MA, Bilir SP. Functional outcomes in head and neck cancer. Semin Radiat Oncol 2004,14,178-89

[63] Kalyankrishna S, Grandis JR. Epidermal growth factor receptor biology in head and neck cancer. J Clin Oncol 2006,24,2666-72

[64] Study of RM-1929 and Photoimmunotherapy in Patients With Recurrent Head and Neck Cancer. ClinicalTrials.gov, 2020. (Accessed February 26, 2021, at <https://clinicaltrials.gov/ct2/show/NCT02422979>.)

[65] Chen J, Zhou J, Lu J, Xiong H, Shi X, Gong L. Significance of CD44 expression in head and neck cancer: a systemic review and meta-analysis. BMC Cancer 2014,14,15

[66] Alexander BM, Cloughesy TF. Adult Glioblastoma. J Clin Oncol 2017,35,2402-9

[67] Omuro A, DeAngelis LM. Glioblastoma and other malignant gliomas: a clinical review. JAMA 2013,310,1842-50

[68] Ostrom QT, Cioffi G, Gittleman H, et al. CBTRUS Statistical Report: Primary Brain and Other Central Nervous System Tumors Diagnosed in the United States in 2012-2016. Neuro Oncol 2019,21,v1-v100

[69] Farina P, Lombardi G, Bergo E, Roma A, Zagonel V. Treatment of malignant gliomas in elderly patients: a concise overview of the literature. Biomed Res Int 2014,2014,734281

[70] Chamberlain MC. Radiographic patterns of relapse in glioblastoma. J Neurooncol 2011,101,319-23

[71] Hochberg FH, Pruitt A. Assumptions in the radiotherapy of glioblastoma. Neurology 1980,30,907-11

[72] Wallner KE, Galicich JH, Krol G, Arbit E, Malkin MG. Patterns of failure following treatment for glioblastoma multiforme and anaplastic astrocytoma. International Journal of Radiation Oncology*Biology*Physics 1989,16,1405-9

[73] Petrecca K, Guiot MC, Panet-Raymond V, Souhami L. Failure pattern following complete resection plus radiotherapy and temozolomide is at the resection margin in patients with glioblastoma. J Neurooncol 2013,111,19-23

[74] Libermann TA, Razon N, Bartal AD, Yarden Y, Schlessinger J, Soreq H. Expression of epidermal growth factor receptors in human brain tumors. Cancer Res 1984,44,753-60

[75] Libermann TA, Nusbaum HR, Razon N, et al. Amplification, enhanced expression and possible rearrangement of EGF receptor gene in primary human brain tumours of glial origin. Nature 1985,313,144-7

[76] Shinojima N, Tada K, Shiraishi S, et al. Prognostic value of epidermal growth factor receptor in patients with glioblastoma multiforme. Cancer Res 2003,63,6962-70

[77] Burley TA, Maczynska J, Shah A, et al. Near-infrared photoimmunotherapy targeting EGFR-Shedding new light on glioblastoma treatment. Int J Cancer 2018,142,2363-74

[78] Lathia JD, Mack SC, Mulkearns-Hubert EE, Valentim CLL, Rich JN. Cancer stem cells in glioblastoma. Genes & development 2015,29,1203-17

[79] Chen R, Nishimura MC, Bumbaca SM, et al. A hierarchy of self-renewing tumor-initiating cell types in glioblastoma. Cancer Cell 2010,17,362-75

[80] Singh SK, Hawkins C, Clarke ID, et al. Identification of human brain tumour initiating cells. Nature 2004,432,396-401

[81] Bao S, Wu Q, Sathornsumetee S, et al. Stem cell-like glioma cells promote tumor angiogenesis through vascular endothelial growth factor. Cancer Res 2006,66,7843-8

[82] Smyth EC, Lagergren J, Fitzgerald RC, et al. Oesophageal cancer. Nat Rev Dis Primers 2017,3,17048

[83] Mukaida H, Toi M, Hirai T, Yamashita Y, Toge T. Clinical significance of the expression of epidermal growth factor and its receptor in esophageal cancer. Cancer 1991,68,142-8

[84] Itakura Y, Sasano H, Shiga C, et al. Epidermal growth factor receptor overexpression in esophageal carcinoma. An immunohistochemical study correlated with clinicopathologic findings and DNA amplification. Cancer 1994,74,795-804

[85] Wang KL, Wu TT, Choi IS, et al. Expression of epidermal growth factor receptor in esophageal and esophagogastric junction adenocarcinomas: association with poor outcome. Cancer 2007,109,658-67

[86] Navarini D, Gurski RR, Madalosso CA, Aita L, Meurer L, Fornari F. Epidermal growth factor receptor expression in esophageal adenocarcinoma: relationship with tumor stage and survival after esophagectomy. Gastroenterol Res Pract 2012,2012,941954

[87] Yacoub L, Goldman H, Odze RD. Transforming growth factor-alpha, epidermal growth factor receptor, and MiB-1 expression in Barrett's-associated neoplasia: correlation with prognosis. Modern pathology : an official journal of the United States and Canadian Academy of Pathology, Inc 1997,10,105-12

[88] Zhan N, Dong WG, Tang YF, Wang ZS, Xiong CL. Analysis of HER2 gene amplification and protein expression in esophageal squamous cell carcinoma. Med Oncol 2012,29,933-40

[89] Van Cutsem E, Bang YJ, Feng-Yi F, et al. HER2 screening data from ToGA: targeting HER2 in gastric and gastroesophageal junction cancer. Gastric Cancer 2015,18,476-84

[90] Cancer Genome Atlas Research N, Analysis Working Group: Asan U, Agency BCC, et al. Integrated genomic characterization of oesophageal carcinoma. Nature 2017,541,169-75

[91] Hartmans E, Linssen MD, Sikkens C, et al. Tyrosine kinase inhibitor induced growth factor receptor upregulation enhances the efficacy of near-infrared targeted photodynamic therapy in esophageal adenocarcinoma cell lines. Oncotarget 2017,8,29846-56

[92] Kato T, Noma K, Ohara T, et al. Cancer-Associated Fibroblasts Affect Intratumoral CD8(+) and FoxP3(+) T Cells Via IL6 in the Tumor Microenvironment. Clin Cancer Res 2018,24,4820-33

[93] Kashima H, Noma K, Ohara T, et al. Cancer-associated fibroblasts (CAFs) promote the lymph node metastasis of esophageal squamous cell carcinoma. Int J Cancer 2019,144,828-40

[94] Watanabe S, Noma K, Ohara T, et al. Photoimmunotherapy for cancer-associated fibroblasts targeting fibroblast activation protein in human esophageal squamous cell carcinoma. Cancer biology & therapy 2019,1-15

[95] Katsube R, Noma K, Ohara T, et al. Fibroblast activation protein targeted near infrared photoimmunotherapy (NIR PIT) overcomes therapeutic resistance in human esophageal cancer. Sci Rep 2021,11,1693

[96] Duma N, Santana-Davila R, Molina JR. Non-Small Cell Lung Cancer: Epidemiology, Screening, Diagnosis, and Treatment. Mayo Clin Proc 2019,94,1623-40

[97] Herbst RS. Review of epidermal growth factor receptor biology. Int J Radiat Oncol Biol Phys 2004,59,21-6

[98] Ansell SM, Lesokhin AM, Borrello I, et al. PD-1 blockade with nivolumab in relapsed or refractory Hodgkin's lymphoma. The New England journal of medicine 2015,372,311-9

[99] Brahmer JR, Tykodi SS, Chow LQ, et al. Safety and activity of anti-PD-L1 antibody in patients with advanced cancer. The New England journal of medicine 2012,366,2455-65

[100] Herbst RS, Soria JC, Kowanetz M, et al. Predictive correlates of response to the anti-PD-L1 antibody MPDL3280A in cancer patients. Nature 2014,515,563-7

[101] Nagaya T, Nakamura Y, Sato K, et al. Near infrared photoimmunotherapy with avelumab, an anti-programmed death-ligand 1 (PD-L1) antibody. Oncotarget 2017,8,8807-17

[102] William WN, Jr., Glisson BS. Novel strategies for the treatment of small-cell lung carcinoma. Nature reviews Clinical oncology 2011,8,611-9

[103] Saunders LR, Bankovich AJ, Anderson WC, et al. A DLL3-targeted antibody-drug conjugate eradicates high-grade pulmonary neuroendocrine tumor-initiating cells in vivo. Science translational medicine 2015,7,302ra136

[104] Isobe Y, Sato K, Nishinaga Y, et al. Near infrared photoimmunotherapy targeting DLL3 for small cell lung cancer. EBioMedicine 2020,52,102632

[105] Mordant P, Arame A, Foucault C, Dujon A, Le Pimpec Barthes F, Riquet M. Surgery for metastatic pleural extension of non-small-cell lung cancer. Eur J Cardiothorac Surg 2011,40,1444-9

[106] Bibby AC, Tsim S, Kanellakis N, et al. Malignant pleural mesothelioma: an update on investigation, diagnosis and treatment. Eur Respir Rev 2016,25,472-86

[107] Schacht V, Ramirez MI, Hong YK, et al. T1alpha/podoplanin deficiency disrupts normal lymphatic vasculature formation and causes lymphedema. Embo j 2003,22,3546-56

[108] Quintanilla M, Montero-Montero L, Renart J, Martin-Villar E. Podoplanin in Inflammation and Cancer. Int J Mol Sci 2019,20

[109] Chuang WY, Chang YS, Yeh CJ, Wu YC, Hsueh C. Role of podoplanin expression in squamous cell carcinoma of upper aerodigestive tract. Histol Histopathol 2013,28,293-9

[110] Nishinaga Y, Sato K, Yasui H, et al. Targeted Phototherapy for Malignant Pleural Mesothelioma: Near-Infrared Photoimmunotherapy Targeting Podoplanin. Cells 2020,9

[111] Ordóñez NG. Application of mesothelin immunostaining in tumor diagnosis. Am J Surg Pathol 2003,27,1418-28

[112] Hassan R, Ho M. Mesothelin targeted cancer immunotherapy. Eur J Cancer 2008,44,46-53

[113] Nagaya T, Nakamura Y, Sato K, et al. Near infrared photoimmunotherapy with an anti-mesothelin antibody. Oncotarget 2016,7,23361-9

[114] Waks AG, Winer EP. Breast Cancer Treatment: A Review. JAMA 2019,321,288-300

[115] Costa RLB, Czerniecki BJ. Clinical development of immunotherapies for HER2(+) breast cancer: a review of HER2-directed monoclonal antibodies and beyond. NPJ Breast Cancer 2020,6,10

[116] Salvador F, Llorente A, Gomis RR. From latency to overt bone metastasis in breast cancer: potential for treatment and prevention. J Pathol 2019,249,6-18

[117] Livraghi L, Garber JE. PARP inhibitors in the management of breast cancer: current data and future prospects. BMC Med 2015,13,188

[118] Shimoyama K, Kagawa S, Ishida M, et al. Viral transduction of the HER2-extracellular domain expands trastuzumab-based photoimmunotherapy for HER2-negative breast cancer cells. Breast Cancer Res Treat 2015,149,597-605

[119] Burness ML, Grushko TA, Olopade OI. Epidermal Growth Factor Receptor in Triple-Negative and Basal-Like Breast Cancer: Promising Clinical Target or Only a Marker? The Cancer Journal 2010,16

[120] Changavi AA, Shashikala A, Ramji AS. Epidermal Growth Factor Receptor Expression in Triple Negative and Nontriple Negative Breast Carcinomas. J Lab Physicians 2015,7,79-83

[121] Nagaya T, Sato K, Harada T, Nakamura Y, Choyke PL, Kobayashi H. Near Infrared Photoimmunotherapy Targeting EGFR Positive Triple Negative Breast Cancer: Optimizing the Conjugate-Light Regimen. PLoS One 2015,10,e0136829

[122] Ajani JA, Lee J, Sano T, Janjigian YY, Fan D, Song S. Gastric adenocarcinoma. Nat Rev Dis Primers 2017,3,17036

[123] Bang YJ, Van Cutsem E, Feyereislova A, et al. Trastuzumab in combination with chemotherapy versus chemotherapy alone for treatment of HER2-positive advanced gastric or gastro-oesophageal junction cancer (ToGA): a phase 3, open-label, randomised controlled trial. Lancet 2010,376,687-97

[124] Sato K, Choyke PL, Kobayashi H. Photoimmunotherapy of gastric cancer peritoneal carcinomatosis in a mouse model. PLoS One 2014,9,e113276

[125] Ito K, Mitsunaga M, Arihiro S, et al. Molecular targeted photoimmunotherapy for HER2-positive human gastric cancer in combination with chemotherapy results in improved treatment outcomes through different cytotoxic mechanisms. BMC Cancer 2016,16,37

[126] Ito K, Mitsunaga M, Nishimura T, Kobayashi H, Tajiri H. Combination photoimmunotherapy with monoclonal antibodies recognizing different epitopes of human epidermal growth factor receptor 2: an assessment of phototherapeutic effect based on fluorescence molecular imaging. Oncotarget 2016,7,14143-52

[127] Cheng G, Mei Y, Pan X, Liu M, Wu S. Expression of HER2/c-erbB-2, EGFR protein in gastric carcinoma and its clinical significance. Open Life Sciences 2019,14,119-25

[128] Gao M, Liang X-J, Zhang Z-S, Ma W, Chang Z-W, Zhang M-Z. Relationship between expression of EGFR in gastric cancer tissue and clinicopathological features. Asian Pacific Journal of Tropical Medicine 2013,6,260-4

[129] Galizia G, Lieto E, Orditura M, et al. Epidermal growth factor receptor (EGFR) expression is associated with a worse prognosis in gastric cancer patients undergoing curative surgery. World J Surg 2007,31,1458-68

[130] Dekker E, Tanis PJ, Vleugels JLA, Kasi PM, Wallace MB. Colorectal cancer. The Lancet 2019,394,1467-80

[131] Hammarström S. The carcinoembryonic antigen (CEA) family: structures, suggested functions and expression in normal and malignant tissues. Seminars in Cancer Biology 1999,9,67-81

[132] Boucher D, Cournoyer D, Stanners CP, Fuks A. Studies on the control of gene expression of the carcinoembryonic antigen family in human tissue. Cancer Res 1989,49,847-52

[133] Tong G, Xu W, Zhang G, et al. The role of tissue and serum carcinoembryonic antigen in stages I to III of colorectal cancer-A retrospective cohort study. Cancer Med 2018,7,5327-38

[134] Park JW, Chang HJ, Kim BC, Yeo HY, Kim DY. Clinical validity of tissue carcinoembryonic antigen expression as ancillary to serum carcinoembryonic antigen concentration in patients curatively resected for colorectal cancer. Colorectal Dis 2013,15,e503-11

[135] Shirasu N, Yamada H, Shibaguchi H, Kuroki M, Kuroki M. Potent and specific antitumor effect of CEA-targeted photoimmunotherapy. Int J Cancer 2014,135,2697-710

[136] Hollandsworth HM, Amirfakhri S, Filemoni F, et al. Near-infrared photoimmunotherapy is effective treatment for colorectal cancer in orthotopic nude-mouse models. PLoS One 2020,15,e0234643

[137] Heath JK, White SJ, Johnstone CN, et al. The human A33 antigen is a transmembrane glycoprotein and a novel member of the immunoglobulin superfamily. Proc Natl Acad Sci U S A 1997,94,469-74

[138] Wei D, Tao Z, Shi Q, et al. Selective Photokilling of Colorectal Tumors by Near-Infrared Photoimmunotherapy with a GPA33-Targeted Single-Chain Antibody Variable Fragment Conjugate. Mol Pharm 2020,17,2508-17

[139] Huang C-W, Chen Y-T, Tsai H-L, et al. EGFR expression in patients with stage III colorectal cancer after adjuvant chemotherapy and on cancer cell function. Oncotarget 2017,8,114663-76

[140] Liu J, Zhou Q, Xu J, Wang J, Zhang Y. Detection of EGFR expression in patients with colorectal cancer and the therapeutic effect of cetuximab. Journal of BUON : official journal of the Balkan Union of Oncology 2016,21,95-100

[141] Theodoropoulos GE, Karafoka E, Papailiou JG, et al. P53 and EGFR expression in colorectal cancer: a reappraisal of 'old' tissue markers in patients with long follow-up. Anticancer Res 2009,29,785-91

[142] Spano JP, Lagorce C, Atlan D, et al. Impact of EGFR expression on colorectal cancer patient prognosis and survival. Annals of Oncology 2005,16,102-8

[143] Xie YH, Chen YX, Fang JY. Comprehensive review of targeted therapy for colorectal cancer. Signal Transduct Target Ther 2020,5,22

[144] Baumhoer D, Tornillo L, Stadlmann S, Roncalli M, Diamantis EK, Terracciano LM. Glypican 3 expression in human nonneoplastic, preneoplastic, and neoplastic tissues: a tissue microarray analysis of 4,387 tissue samples. Am J Clin Pathol 2008,129,899-906

[145] Hanaoka H, Nagaya T, Sato K, et al. Glypican-3 targeted human heavy chain antibody as a drug carrier for hepatocellular carcinoma therapy. Mol Pharm 2015,12,2151-7

[146] Banales JM, Marin JJG, Lamarca A, et al. Cholangiocarcinoma 2020: the next horizon in mechanisms and management. Nat Rev Gastroenterol Hepatol 2020,17,557-88

[147] Hirata H, Kuwatani M, Nakajima K, et al. Near-infrared photoimmunotherapy (NIR-PIT) on cholangiocarcinoma using a novel catheter device with light emitting diodes. Cancer Sci 2020

[148] Goldenberg DM, Cardillo TM, Govindan SV, Rossi EA, Sharkey RM. Trop-2 is a novel target for solid cancer therapy with sacituzumab govitecan (IMMU-132), an antibody-drug conjugate (ADC). Oncotarget 2015,6,22496-512

[149] Shvartsur A, Bonavida B. Trop2 and its overexpression in cancers: regulation and clinical/therapeutic implications. Genes Cancer 2015,6,84-105

[150] Nishimura T, Mitsunaga M, Sawada R, et al. Photoimmunotherapy targeting biliary-pancreatic cancer with humanized anti-TROP2 antibody. Cancer medicine 2019,8,7781-92

[151] Moletta L, Serafini S, Valmasoni M, Pierobon ES, Ponzoni A, Sperti C. Surgery for Recurrent Pancreatic Cancer: Is It Effective? Cancers (Basel) 2019,11

[152] Maawy AA, Hiroshima Y, Zhang Y, et al. Near infra-red photoimmunotherapy with anti-CEA-IR700 results in extensive tumor lysis and a significant decrease in tumor burden in orthotopic mouse models of pancreatic cancer. PLoS One 2015,10,e0121989

[153] Lum YL, Luk JM, Staunton DE, Ng DKP, Fong WP. Cadherin-17 Targeted Near-Infrared Photoimmunotherapy for Treatment of Gastrointestinal Cancer. Mol Pharm 2020,17,3941-51

[154] Hiroshima Y, Maawy A, Zhang Y, et al. Photoimmunotherapy Inhibits Tumor Recurrence After Surgical Resection on a Pancreatic Cancer Patient-Derived Orthotopic Xenograft (PDOX) Nude Mouse Model. Ann Surg Oncol 2015,22 Suppl 3,S1469-74

[155] Maawy AA, Hiroshima Y, Zhang Y, et al. Photoimmunotherapy lowers recurrence after pancreatic cancer surgery in orthotopic nude mouse models. J Surg Res 2015,197,5-11

[156] Park SJ, Gu MJ, Lee DS, Yun SS, Kim HJ, Choi JH. EGFR expression in pancreatic intraepithelial neoplasia and ductal adenocarcinoma. Int J Clin Exp Pathol 2015,8,8298-304

[157] Handra-Luca A, Hammel P, Sauvanet A, Lesty C, Ruszniewski P, Couvelard A. EGFR expression in pancreatic adenocarcinoma. Relationship to tumour morphology and cell adhesion proteins. J Clin Pathol 2014,67,295-300

[158] Bloomston M, Bhardwaj A, Ellison EC, Frankel WL. Epidermal growth factor receptor expression in pancreatic carcinoma using tissue microarray technique. Digestive surgery 2006,23,74-9

[159] Reid BM, Permuth JB, Sellers TA. Epidemiology of ovarian cancer: a review. Cancer Biol Med 2017,14,9-32

[160] Matulonis UA, Sood AK, Fallowfield L, Howitt BE, Sehouli J, Karlan BY. Ovarian cancer. Nat Rev Dis Primers 2016,2,16061

[161] Horowitz NS, Miller A, Rungruang B, et al. Does aggressive surgery improve outcomes? Interaction between preoperative disease burden and complex surgery in patients with advanced-stage ovarian cancer: an analysis of GOG 182. J Clin Oncol 2015,33,937-43

[162] Chang SJ, Hodeib M, Chang J, Bristow RE. Survival impact of complete cytoreduction to no gross residual disease for advanced-stage ovarian cancer: a meta-analysis. Gynecol Oncol 2013,130,493-8

[163] Pomel C, Jeyarajah A, Oram D, et al. Cytoreductive surgery in ovarian cancer. Cancer Imaging 2007,7,210-5

[164] Sato K, Hanaoka H, Watanabe R, Nakajima T, Choyke PL, Kobayashi H. Near infrared photoimmunotherapy in the treatment of disseminated peritoneal ovarian cancer. Mol Cancer Ther 2015,14,141-50

[165] Hama Y, Urano Y, Koyama Y, et al. In vivo spectral fluorescence imaging of submillimeter peritoneal cancer implants using a lectin-targeted optical agent. Neoplasia 2006,8,607-12

[166] Harada T, Nakamura Y, Sato K, et al. Near-infrared photoimmunotherapy with galactosyl serum albumin in a model of diffuse peritoneal disseminated ovarian cancer. Oncotarget 2016,7,79408-16

[167] Sanli O, Dobruch J, Knowles MA, et al. Bladder cancer. Nat Rev Dis Primers 2017,3,17022

[168] Chow NH, Chan SH, Tzai TS, Ho CL, Liu HS. Expression profiles of ErbB family receptors and prognosis in primary transitional cell carcinoma of the urinary bladder. Clin Cancer Res 2001,7,1957-62

[169] Railkar R, Krane LS, Li QQ, et al. Epidermal Growth Factor Receptor (EGFR)-targeted Photoimmunotherapy (PIT) for the Treatment of EGFR-expressing Bladder Cancer. Molecular cancer therapeutics 2017,16,2201-14

[170] Nagaya T, Okuyama S, Ogata F, et al. Near infrared photoimmunotherapy targeting bladder cancer with a canine anti-epidermal growth factor receptor (EGFR) antibody. Oncotarget 2018,9,19026-38

[171] Siddiqui MR, Railkar R, Sanford T, et al. Targeting Epidermal Growth Factor Receptor (EGFR) and Human Epidermal Growth Factor Receptor 2 (HER2) Expressing Bladder Cancer Using Combination Photoimmunotherapy (PIT). Sci Rep 2019,9,2084

[172] Chan KS, Espinosa I, Chao M, et al. Identification, molecular characterization, clinical prognosis, and therapeutic targeting of human bladder tumor-initiating cells. Proc Natl Acad Sci U S A 2009,106,14016-21

[173] Kiss B, van den Berg NS, Ertsey R, et al. CD47-Targeted Near-infrared Photoimmunotherapy for Human Bladder Cancer. Clin Cancer Res 2019

[174] Haberkorn U, Eder M, Kopka K, Babich JW, Eisenhut M. New Strategies in Prostate Cancer: Prostate-Specific Membrane Antigen (PSMA) Ligands for Diagnosis and Therapy. Clin Cancer Res 2016,22,9-15

[175] Nagaya T, Nakamura Y, Okuyama S, et al. Near-Infrared Photoimmunotherapy Targeting Prostate Cancer with Prostate-Specific Membrane Antigen (PSMA) Antibody. Mol Cancer Res 2017,15,1153-62

[176] Watanabe R, Hanaoka H, Sato K, et al. Photoimmunotherapy targeting prostate-specific membrane antigen: are antibody fragments as effective as antibodies? J Nucl Med 2015,56,140-4

[177] Bouchelouche K, Turkbey B, Choyke PL. PSMA PET and Radionuclide Therapy in Prostate Cancer. Seminars in nuclear medicine 2016,46,522-35

[178] Sabattini E, Bacci F, Sagramoso C, Pileri SA. WHO classification of tumours of haematopoietic and lymphoid tissues in 2008: an overview. Pathologica 2010,102,83-7

[179] Chung C. Current targeted therapies in lymphomas. Am J Health Syst Pharm 2019,76,1825-34

[180] Nagaya T, Nakamura Y, Sato K, Harada T, Choyke PL, Kobayashi H. Near infrared photoimmunotherapy of B-cell lymphoma. Mol Oncol 2016,10,1404-14

[181] Heryanto YD, Hanaoka H, Nakajima T, Yamaguchi A, Tsushima Y. Applying near-infrared photoimmunotherapy to B-cell lymphoma: comparative evaluation with radioimmunotherapy in tumor xenografts. Ann Nucl Med 2017,31,669-77

[182] Al Hothali GI. Review of the treatment of mycosis fungoides and Sézary syndrome: A stage-based approach. Int J Health Sci (Qassim) 2013,7,220-39

[183] Magro CM, Dyrsen ME. Cutaneous lymphocyte antigen expression in benign and neoplastic cutaneous B- and T-cell lymphoid infiltrates. J Cutan Pathol 2008,35,1040-9

[184] Silic-Benussi M, Saponeri A, Michelotto A, et al. Near infrared photoimmunotherapy targeting the cutaneous lymphocyte antigen for mycosis fungoides. Expert Opin Biol Ther 2020,1-5

[185] Domingues B, Lopes JM, Soares P, Populo H. Melanoma treatment in review. Immunotargets Ther 2018,7,35-49

[186] Dye DE, Medic S, Ziman M, Coombe DR. Melanoma biomolecules: independently identified but functionally intertwined. Front Oncol 2013,3,252

[187] Wei W, Jiang D, Ehlerding EB, et al. CD146-Targeted Multimodal Image-Guided Photoimmunotherapy of Melanoma. Adv Sci (Weinh) 2019,6,1801237

[188] Macedo F, Ladeira K, Pinho F, et al. Bone Metastases: An Overview. Oncol Rev 2017,11,321

[189] Nakamura YA, Okuyama S, Furusawa A, et al. Near-infrared photoimmunotherapy through bone. Cancer Sci 2019,110,3689-94

**Figure 1 The mechanisms of cell death caused by NIR-PIT.**

(A) Structural change of IR700. Upon NIR light exposure, axial ligands are released from the IR700 molecule. Adapted from Ref. [8]. (B) Scheme of the cell killing mechanism induced by NIR-PIT. An antibody-IR700-antigen complex is formed on the antigen on the cell membrane. The conformational change of conjugate produces physical stress in the cell membrane, resulting in the weakening and rupture of the cell membrane. The water outside of the cell is flown into the cell, leading cell death. Adapted from Ref. [8].

**Figure 2 The mechanisms of SUPR effects induced by NIR-PIT.**

(A) Scheme of SUPR effect induced by NIR-PIT. Many of the initial cell killing occurs in the perivascular layer of tumor cells after NIR-PIT, leading form the potential space around the tumor vasculature. It increases vascular permeability and decreases interstitial pressures. Then, nanodrug delivery to the remain tumor can be enhanced. Adapted from Ref. [8].

(B) The increases of pegylated quantum dot 800 into tumor bed 1 hour after NIR-PIT were observed compared to control tumors (up to 24-fold). Adapted from Ref. [8].

**Figure 3 Various cancers and promising targets for NIR-PIT.**
